# Mutant p53 sensitizes KRAS-driven lung adenocarcinoma to immunotherapy by repressing SPP1

**DOI:** 10.1126/sciadv.aeg9953

**Published:** 2026-09-11

**Authors:** Xin Zhang, Qian Hao, Wenyue Yang, Yunbo Yu, Qingya Yan, Zhenhua Wu, Zhihuang Hu, Xiang Zhou, Jialei Wang

**Affiliations:** ^1^ Department of Medical Oncology, Fudan University Shanghai Cancer Center, Shanghai 200032, China.; ^2^ Department of Oncology, Shanghai Medical College, Fudan University, Shanghai, China.; ^3^ Institute of Thoracic Oncology, Fudan University Shanghai Cancer Center, Shanghai 200032, China.; ^4^ Cancer Institute, Fudan University Shanghai Cancer Center, Shanghai 200032, China.; ^5^ Key Laboratory of Breast Cancer in Shanghai, Department of Breast Surgery, Fudan University Shanghai Cancer Center, Shanghai 200032, China.

## Abstract

The efficacy of immune checkpoint blockade (ICB) in lung adenocarcinoma (LUAD) is limited by primary or acquired resistance, especially in genetically defined subsets. We investigated the impact of *KRAS/TP53* co-mutation on ICB response. Our analysis reveals that *KRAS/TP53* co-mutation is associated with favorable ICB outcomes, a phenotype orchestrated by secreted phosphoprotein 1 (SPP1). Mechanistically, mutant p53 and *KRAS* exert opposing effects on SPP1. Mutant p53 synergizes with transcription factor FOXA1 to repress *SPP1* transcription, whereas *KRAS* activates the mitogen-activated protein kinase (MAPK)–extracellular signal–regulated kinase (ERK)–signal transducer and activator of transcription 1 (STAT1) axis to up-regulate *SPP1*. Consequently, reduced SPP1 level in *KRAS/TP53* co-mutant LUAD impairs myeloid-derived suppressor cell (MDSC) recruitment and increases CD8+ T cell infiltration. Notably, SPP1 blockade synergizes with anti–programmed death-1 (PD-1) therapy to suppress tumor growth in *KRAS*-mutant xenograft models. Collectively, we delineate a mutant p53–FOXA1–SPP1 regulatory axis that modulates ICB susceptibility and unveil a synergistic combination therapy strategy.

## INTRODUCTION

Lung adenocarcinoma (LUAD), the most prevalent subtype of lung cancer, remains a leading cause of cancer-related mortality worldwide (*1*). The advent of immune checkpoint blockade (ICB), particularly those targeting programmed death-1 (PD-1) and programmed death-ligand 1 (PD-L1), has improved survival for patients with advanced LUAD (*2*–*4*). However, therapeutic efficacy is substantially limited by resistance. Approximately 30 to 40% of patients exhibit primary resistance, whereas many initial responders later develop acquired resistance with disease progression within 12 to 18 months (*5*, *6*). Consequently, only about 20% of patients with advanced non–small cell lung cancer (NSCLC) achieve long-term benefits from ICB (*7*). These challenges underscore the urgent need to elucidate the mechanisms underlying immunotherapy resistance and to develop rational combination strategies for specific LUAD subtypes.

*KRAS* mutation is one of the most prevalent driver alterations in NSCLC, occurring in ∼20 to 30% of cases (*8*). Although recent breakthroughs in *KRAS*-targeted therapy have provided more options for pretreated patients, first-line treatment for advanced *KRAS*-mutant LUAD still relies on ICB-based combination therapy, following the paradigm for driver mutation-negative NSCLC (*9*, *10*). Optimizing ICB efficacy in this population is therefore critically important. Clinical responses to ICB in *KRAS*-mutant LUAD are highly heterogeneous, a variability strongly influenced by co-occurring genetic alterations (*11*, *12*). Among these, concurrent *TP53* mutation is the most common (*13*). As a classic tumor suppressor gene, wild-type p53 maintains genomic stability by regulating cell cycle arrest, DNA repair, senescence, and apoptosis (*14*). However, *TP53* mutations are common in lung cancer. Missense mutations within the DNA binding domain (DBD) abrogate DNA binding capacity and disrupt the transcriptional regulatory functions of wild-type p53. In addition, mutant p53 often acquires dominant-negative effects or gain-of-function (GOF) properties (*15*). Recent studies suggested that mutant p53 could modulate the tumor microenvironment (TME). In NSCLC, mutant p53 has been implicated in both suppressing innate immune signals and shaping adaptive immunity, correlating with features such as elevated PD-L1 expression, increased tumor mutation burden (TMB), and enhanced CD8+ T cell infiltration (*16*, *17*). Despite these insights, previous studies have yielded conflicting conclusions regarding the combined impact of *KRAS/TP53* co-mutations on ICB efficacy in LUAD (*13*, *18*, *19*), and the underlying molecular mechanisms remain poorly delineated, hindering patient stratification and the development of tailored combination therapies.

Secreted phosphoprotein 1 (SPP1) is a key extracellular matrix glycoprotein that orchestrates immunosuppressive TME remodeling (*20*). Several studies have demonstrated that SPP1 promotes tumor stemness via the CD44-ERK axis and drives macrophage polarization toward an immunosuppressive M2-like phenotype (*21*). Preclinical studies further show that targeting SPP1 enhances anti–PD-1 efficacy by increasing cytotoxic T cell infiltration and reducing cancer-associated fibroblasts (CAFs). In addition, SPP1 facilitates immune evasion by recruiting myeloid-derived suppressor cells (MDSCs) (*22*, *23*). Despite its pivotal role in these processes, the relationship between SPP1 and gene mutational status remains largely unexplored.

In this study, we first validated the association between *KRAS/TP53* co-mutations and improved ICB outcomes in patients with LUAD. We then identified SPP1, a key immunoregulator in the TME, as a critical downstream effector comodulated by mutant p53 and mutant *KRAS*. These two mutations exert opposing effects on SPP1 expression. Mechanistically, mutant p53 cooperates with the transcription factor (TF) *FOXA1* to repress *SPP1* transcription, whereas oncogenic *KRAS* up-regulates SPP1 by activating the mitogen-activated protein kinase (MAPK)–extracellular signal–regulated kinase (ERK)–signal transducer and activator of transcription 1 (STAT1) axis. Functionally, in *KRAS*-mutant LUAD, mutant p53 remodels the TME by reducing SPP1 secretion, which impairs monocytic-derived myeloid-derived suppressor cell (M-MDSC) recruitment and enhances CD8+ T cell infiltration. Our results demonstrate that SPP1 blockade synergizes with anti–PD-1 therapy to suppress tumor growth in a p53-null, *KRAS*-mutant xenograft model. Collectively, this study provides a mechanistic insight and an actionable therapeutic strategy for personalized immunotherapy in *KRAS*-driven LUAD.

## RESULTS

### Co-mutation of *KRAS* and *TP53* correlates with favorable prognosis in patients with LUAD receiving immunotherapy

**Fig. 1.**
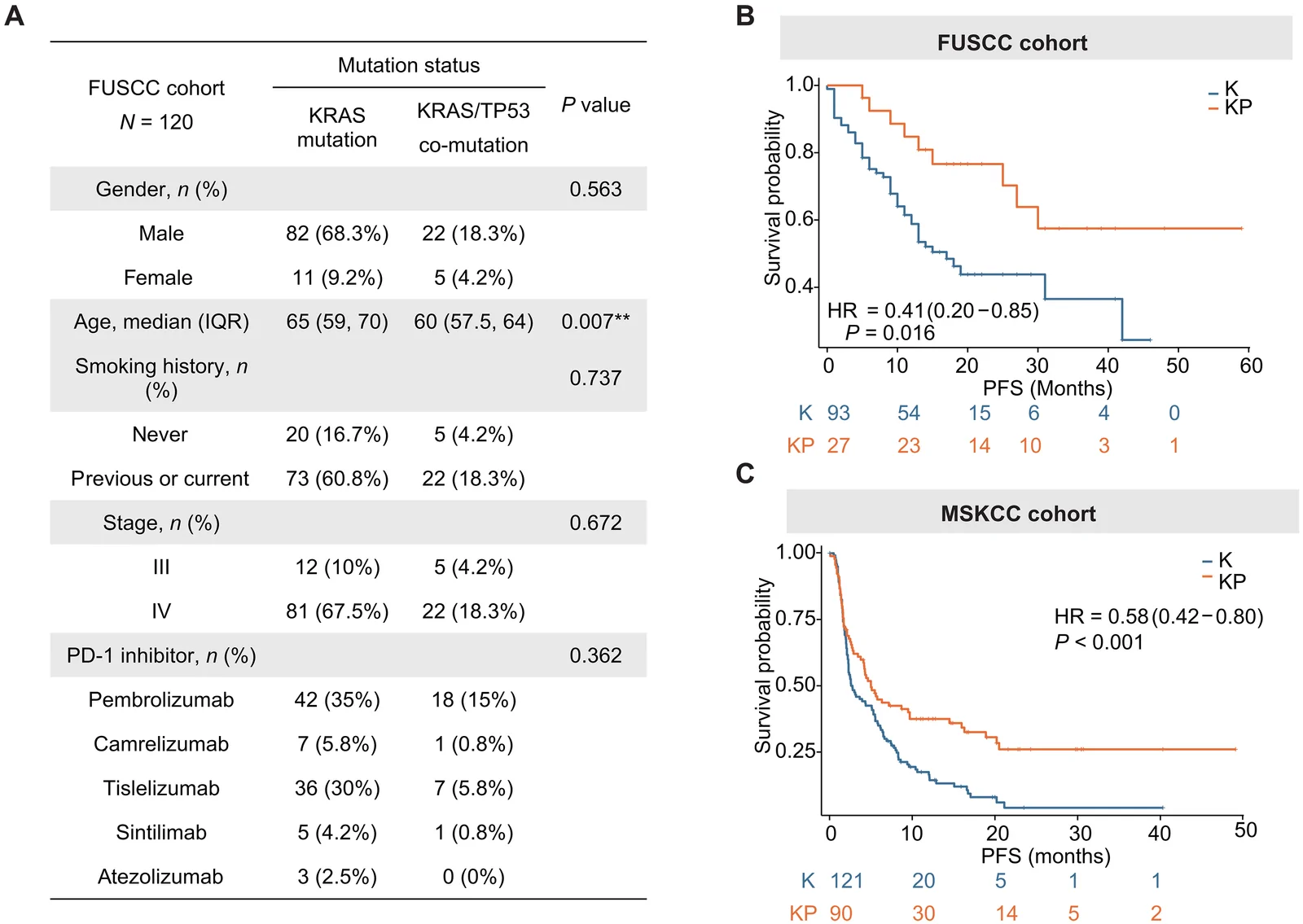
*KRAS/TP53* co-mutation predicts favorable prognosis in immunotherapy treated LUAD. (**A**) Clinical characteristics associated with *KRAS* and *TP53* mutation status in the FUSCC cohort (*n* = 120). Statistical significance was determined using the two-sided chi-square test. IQR, interquartile range. (**B**) Survival analysis of PFS between patients with *KRAS* mutation and *KRAS/TP53* co-mutations in the FUSCC cohort (*n* = 120). Statistical significance was determined using the log-rank test. HR, hazard ratio. (**C**) Survival analysis of PFS between patients with *KRAS* mutation and *KRAS/TP53* co-mutations in the MSKCC cohort (*n* = 211). Statistical significance was determined using the log-rank test.

To investigate the impact of *KRAS* mutation alone and *KRAS/TP53* co-mutations on the efficacy of immunotherapy in patients with LUAD, we retrospectively enrolled 120 patients with advanced LUAD harboring *KRAS* mutation or *KRAS/TP53* co-mutations who received immunotherapy at the FUSCC. Patients in the co-mutation group were younger, whereas no significant differences were observed in gender, smoking history, clinical stage, or PD-1 inhibitor regimen between the two groups (Fig. 1A). Survival analyses indicated that patients with *KRAS/TP53* co-mutations had significantly longer progression-free survival (PFS) and overall survival (OS) compared with those with *KRAS* mutation alone (Fig. 1B and fig. S1A). This finding was validated using data from the MSKCC cohort (Fig. 1C and fig. S1B). In addition, univariate and multivariate Cox regression analyses confirmed that *KRAS/TP53* co-mutation serves as an independent predictor of prognosis (table S1). Notably, we did not observe this survival benefit in a comparable pancreatic cancer immunotherapy cohort (*24*) (fig. S1, C to E). Collectively, these results demonstrate that *KRAS/TP53* co-mutation is associated with favorable prognosis in patients with LUAD treated with immunotherapy.

### Mutant p53 down-regulates SPP1 expression

**Fig. 2.**
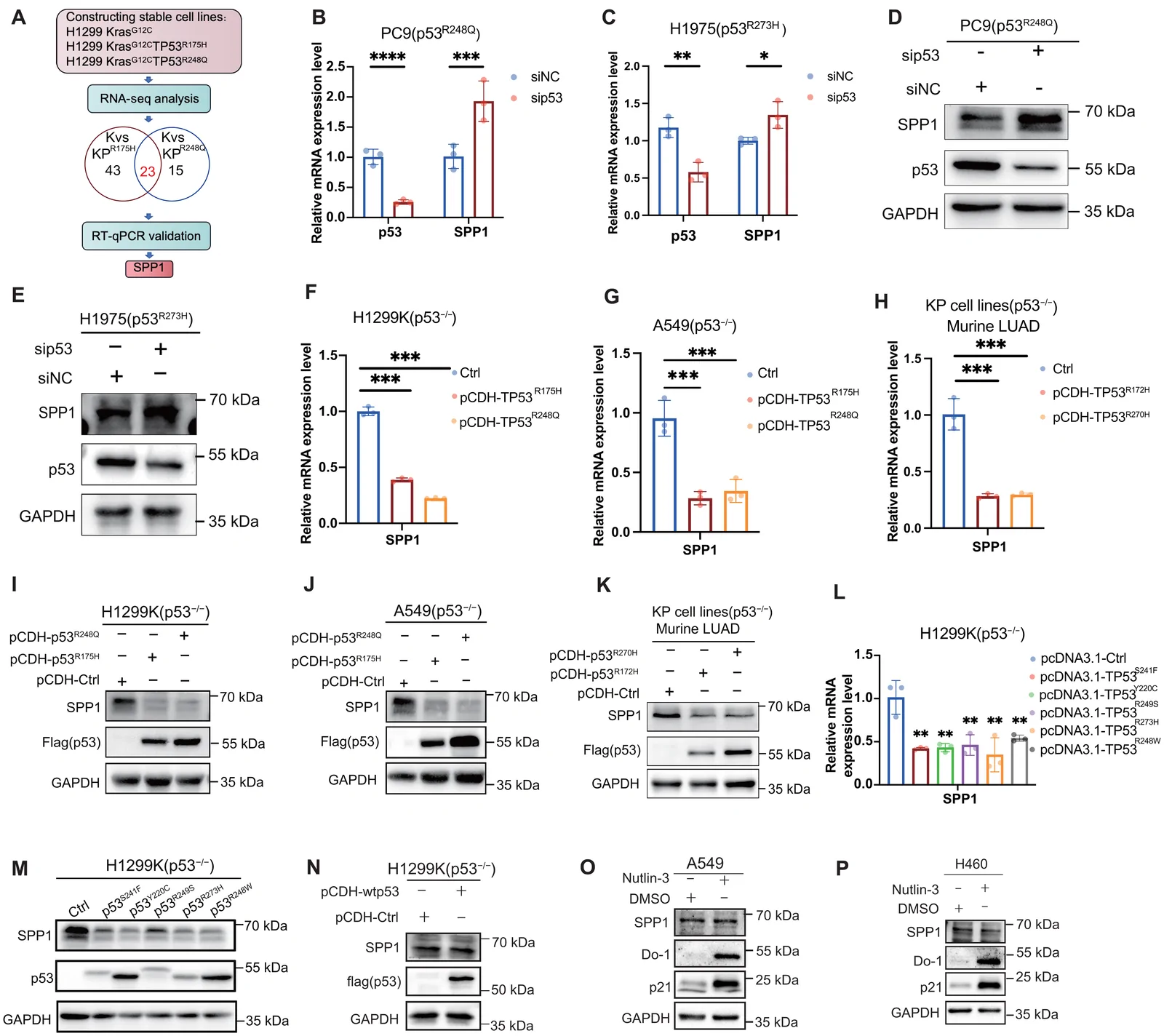
Inhibition of SPP1 expression by mutant p53. (**A**) Schematic diagram depicting the identification of SPP1. (**B** to **E**) RT-qPCR and WB assays validating the expression levels of SPP1 in PC-9 [(B) and(D)] cells and H1975 [(C) and (E)] cells transfected with siNC and sip53. (**F** to **K**) RT-qPCR and WB assays validating the expression levels of SPP1 in H1299K [(F) and (I)] and A549^p53−/−^[(G) and (J)] cells transfected with Flag-Ctrl, Flag-p53-R175H, and Flag-p53-R248Q; KP cells transfected with Flag-Ctrl, Flag-p53-R172H, and Flag-p53-R270H [(H) and (K)]. Data are means ± SD (*n* = 3). **P < 0.01; ***P < 0.001; ****P < 0.0001, two-tailed unpaired *t* test. (**L** and **M**) RT-qPCR (L) and WB (M) assays validating the expression levels of SPP1 in H1299K cells transfected with Flag-Ctrl, Flag-p53-S241F, Flag-p53-Y220C, Flag-p53-R249S, Flag-p53-R273H, and Flag-p53-R248W. Data are means ± SD (*n* = 3). **P < 0.01, one-way ANOVA. (**N**) WB assay validating the expression level of SPP1 in H1299K cells transfected with Flag-Ctrl and Flag-wtp53. (**O** and **P**) WB assay validating the expression level of SPP1 in A549 (O) and H460 (P) cells treated with dimethyl sulfoxide (DMSO) or Nutlin-3 (20 μM).

To elaborate the molecular mechanisms by which mutant p53 modulates downstream gene expression and signaling pathways in the context of *KRAS* mutations, we conducted transcriptome sequencing in H1299 cells harboring KRAS Gly12Cys (G12C) (K), as well as KRAS G12C co-mutated with either p53-Arg175His (R175H) (KP^R175H^) or p53-Arg248Gln (R248Q) (KP^R248Q^) (fig. S2A). Differentially expressed genes (DEGs) were identified from the K versus KP^R175H^ and K versus KP^R248Q^ comparisons using thresholds of fold change (FC) > 2 and adjusted *P* values < 0.05. The intersection of these two DEG sets identified 23 genes commonly regulated by p53-R175H and p53-R248Q (Fig. 2A), Kyoto Encyclopedia of Genes and Genomes (KEGG) pathway enrichment analysis of the DEGs revealed that transcriptional misregulation in cancer was significantly enriched in both groups (fig. S2, B to E). To validate mutant p53–mediated regulation of these candidate genes, we conducted reverse transcription quantitative polymerase chain reaction (RT-qPCR) and confirmed consistent down-regulation of *SPP1* (fig. S2, F to H). Furthermore, transient knockdown of mutant p53 in PC9 and H1975 led to up-regulation of both *SPP1* mRNA and protein levels (Fig. 2, B to E). Conversely, ectopic expression of mutant p53 in H1299K (KRAS^G12C^-mutant H1299 cells), A549^p53−/−^, and KP (KRAS^Gly12Asp (G12D)^/TP53^−/−^ mouse adenocarcinoma cell line) cells resulted in down-regulation of *SPP1* mRNA and protein expression (Fig. 2, F to K). To systematically assess the regulatory effects of different p53 mutants on SPP1 expression, we overexpressed p53-Ser241Phe (S241F), p53-Tyr220Cys (Y220C), p53-Arg249Ser (R249S), p53-Arg273His (R273H), and p53-Arg248Trp (R248W) in H1299K cells. These mutant p53 variants suppressed *SPP1* mRNA and protein expression (Fig. 2, L and M). However, overexpression of wild-type p53 in H1299K cells, pharmacological activation via Nutlin-3, and small interfering RNA (siRNA)–mediated knockdown of endogenous wild-type p53 in A549 and H460 cells consistently failed to alter SPP1 protein levels (Fig. 2, N to P, and fig. S2, K to N). Together, these findings demonstrate that mutant p53 represses *SPP1* expression in a manner independent of its specific mutation site.

### Mutant p53 cooperates with FOXA1 to repress *SPP1* transcription

**Fig. 3.**
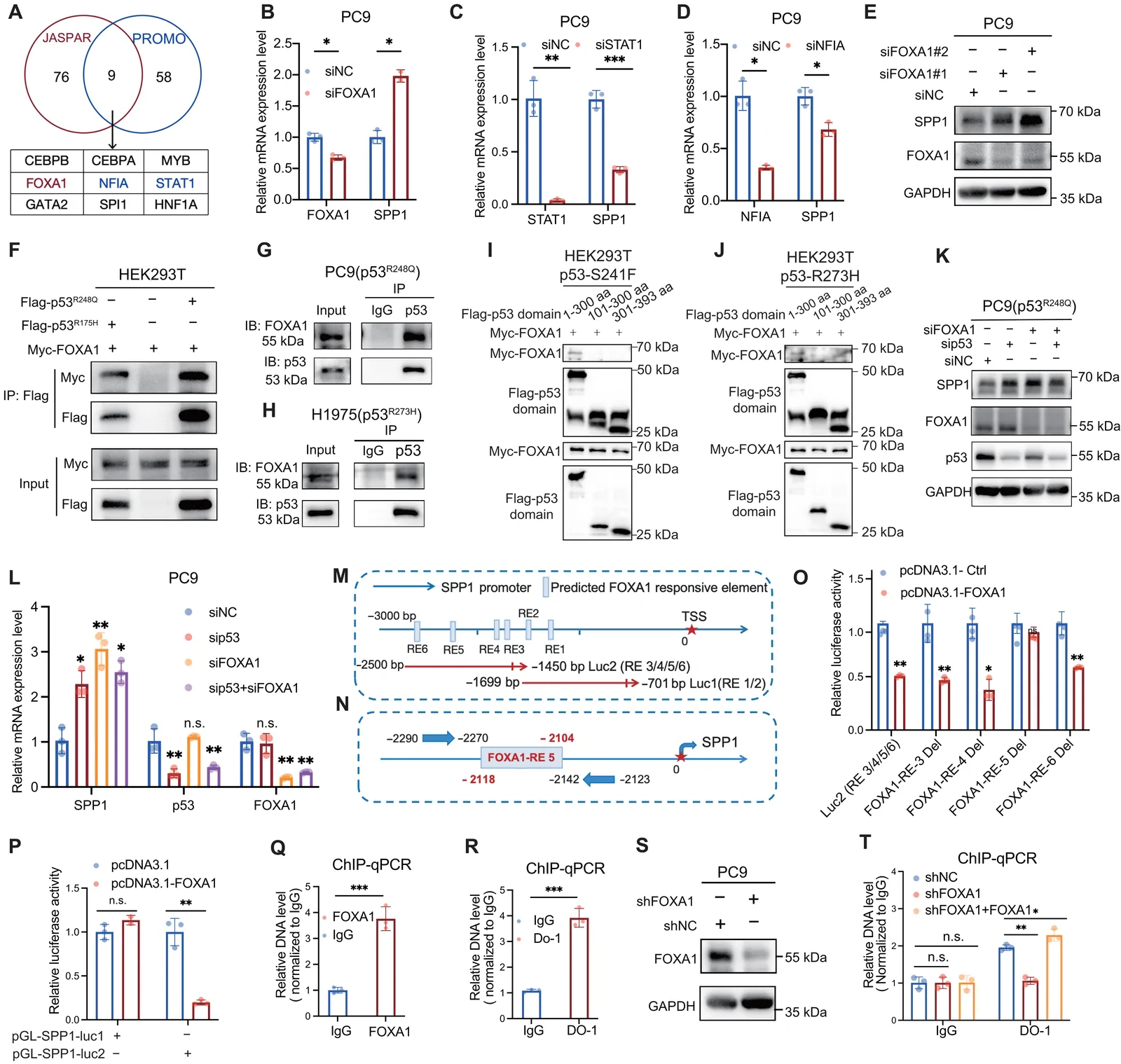
Mutant p53 cooperates with FOXA1 to repress SPP1 transcription. (**A**) Venn diagram of SPP1 candidate TFs predicted by JASPAR and PROMO databases. (**B** to **D**) RT-qPCR analysis of candidate TFs and *SPP1* mRNA levels in PC9 cells transfected with siRNAs targeting *FOXA1* (B), *STAT1* (C), or *NFIA* (D). Data are means ± SD (*n* = 3). *P < 0.05; **P < 0.01; ***P < 0.001, two-tailed unpaired *t* test. (**E**) Western blot analysis of *FOXA1* and *SPP1* protein levels in PC9 cells transfected with two independent siRNAs targeting *FOXA1* (siFOXA1#1 and siFOXA1#2). (**F**) Exogenous co-IP assay in HEK293T cells cotransfected with Flag-tagged control plasmid or Flag-tagged mutant p53 (p53-R175H or p53-R248Q) and Myc-tagged FOXA1. Input: total cell lysate; IP: immunoprecipitation with anti-Flag antibody. Western blot (IB) was performed with anti-Myc or anti-Flag antibody. (**G** and **H**) Endogenous co-IP assay in PC9 [p53-R248Q mutant; (F)] and H1975 [p53-R273H mutant; (G)] cells. Endogenous FOXA1 was immunoprecipitated (IP: anti-FOXA1), and coprecipitated mutant p53 was detected by IB (anti-p53). IgG: isotype control. (**I** and **J**) Mapping experiments in HEK293T cell cotransfected with Flag-tagged mutant p53 domain and Myc-tagged FOXA1. Input: total cell lysate; IP: immunoprecipitation with anti-Flag antibody. Western blot (IB) was performed with anti-Myc or anti-Flag antibody. aa, amino acids. (**K** and **L**) Western blot (K) and RT-qPCR (L) analysis of SPP1, p53, and FOXA1 mRNA and protein levels in PC9 cells transfected with siNC, sip53, siFOXA1, or sip53+siFOXA1. Data are means ± SD (*n* = 3). *P < 0.05; **P < 0.01; n.s., not significant, two-tailed unpaired *t* test. (**M** and **N**) Schematic of the *SPP1* promoter region. Predicted FOXA1 REs in the *SPP1* promoter (M) and amplicon region [−2390 to −2115 base pairs (bp)] containing FOXA1-RE 5 used for ChIP assays (N). (**O**) Dual-luciferase reporter assay in PC9 cells transfected with pcDNA3.1-FOXA1 and pGL3-SPP1-luc. Relative luciferase activity (normalized to Renilla) was measured. Data are means ± SD (*n* = 3). **P < 0.01; n.s., not significant, two-tailed unpaired *t* test. (**P**) Dual-luciferase reporter assay in PC9 cells cotransfected with pGL-SPP1-luc2 promoter luciferase and FOXA1-RE 3/4/5/6 deletion constructs and pcDNA3.1-FOXA1. Relative luciferase activity (normalized to Renilla) was measured. Data are means ± SD (*n* = 3). *P < 0.05; **P < 0.01; n.s., not significant, two-tailed unpaired *t* test. (**Q** and **R**) ChIP-qPCR analysis of *FOXA1* (Q) and p53 [(R); detected by Do-1 antibody] recruitment on the *SPP1* promoter region (FOXA1-RE 5). Data are means ± SD (*n* = 3). ***P < 0.001, two-tailed unpaired *t* test. (**S**) Western blot analysis of FOXA1 protein levels in PC9 cells transfected with shFOXA1 or shNC. (**T**) ChIP-qPCR analysis of p53 recruitment on the *SPP1* promoter region (FOXA1-RE 5) in PC9 cells transfected with shNC, shFOXA1, or shFOXA1+FOXA1. *P < 0.05; **P < 0.01; n.s., not significant, one-way ANOVA.

Mutant p53 modulates gene transcription via interactions with TFs or cofactors (*15*). To explore how mutant p53 regulates SPP1, we used JASPAR and PROMO databases to predict candidate TFs regulating SPP1. Intersecting the results yielded nine candidate TFs: *CEBPB*, *CEBPA*, *MYB*, *FOXA1*, *NFIA*, *STAT1*, *GATA2*, *SPI1*, and *HNF1A* (Fig. 3A). We then knocked down each of these candidate TFs and found that *FOXA1*, *NFIA*, and *STAT1* are key SPP1 regulators (Fig. 3, B to D, and fig. S3, A to F). Given that mutant p53 exerts its functions via interactions with TFs, we further performed coimmunoprecipitation (co-IP) assays and found that only FOXA1 specifically interacted with mutant p53 (Fig. 3, F to H, and fig. S3, G and H). Mapping experiments revealed that FOXA1 specifically interacts with the N-terminal domain (1 to 100 amino acids) of mutant p53 (Fig. 3, I and J). In addition, knockdown of *FOXA1* increased *SPP1* mRNA and protein levels, and TCGA-LUAD data confirmed a negative correlation between the expression of *SPP1* and *FOXA1*, suggesting that *FOXA1* is an inhibitory TF of SPP1 (Fig. 3E and fig. S3, I and J). Notably, knockdown of mutant p53 did not affect *FOXA1* expression (fig. S3, K to N). Furthermore, single knockdown of mutant p53 or FOXA1 up-regulated SPP1, whereas co-knockdown did not enhance this effect, indicating that mutant p53 regulates SPP1 expression through its interaction with FOXA1 (Fig. 3, K to L). To further validate this observation, we used the JASPAR database to analyze the genomic sequence upstream of the transcription start site (TSS) of *SPP1*, and identified six possible FOXA1 response elements (REs) (Fig. 3M). The dual luciferase reporter assay and chromatin immunoprecipitation quantitative polymerase chain reaction (ChIP-qPCR) were conducted to verify that FOXA1 specifically binds to RE 5, which is located at −2118 to −2104 upstream of the *SPP1* TSS, to inhibit the *SPP1* promoter-driven luciferase activity (Fig. 3, N to P). Furthermore, we tested whether FOXA1 influences the recruitment of mutant p53 to the SPP1 promoter through the ChIP-qPCR assay. Our results showed that *FOXA1* depletion reduced the association of mutant p53 in the *SPP1* promoter, whereas the overexpression of FOXA1 increased mutant p53 binding to *SPP1* promoter (Fig. 3, Q to T). Collectively, our results demonstrate that mutant p53 binds to the *SPP1* promoter indirectly by interacting with FOXA1, which is dependent on FOXA1’s DNA binding capacity, to cooperatively repress *SPP1* transcription.

### Mutant p53 impairs monocyte migration by reducing SPP1 secretion

**Fig. 4.**
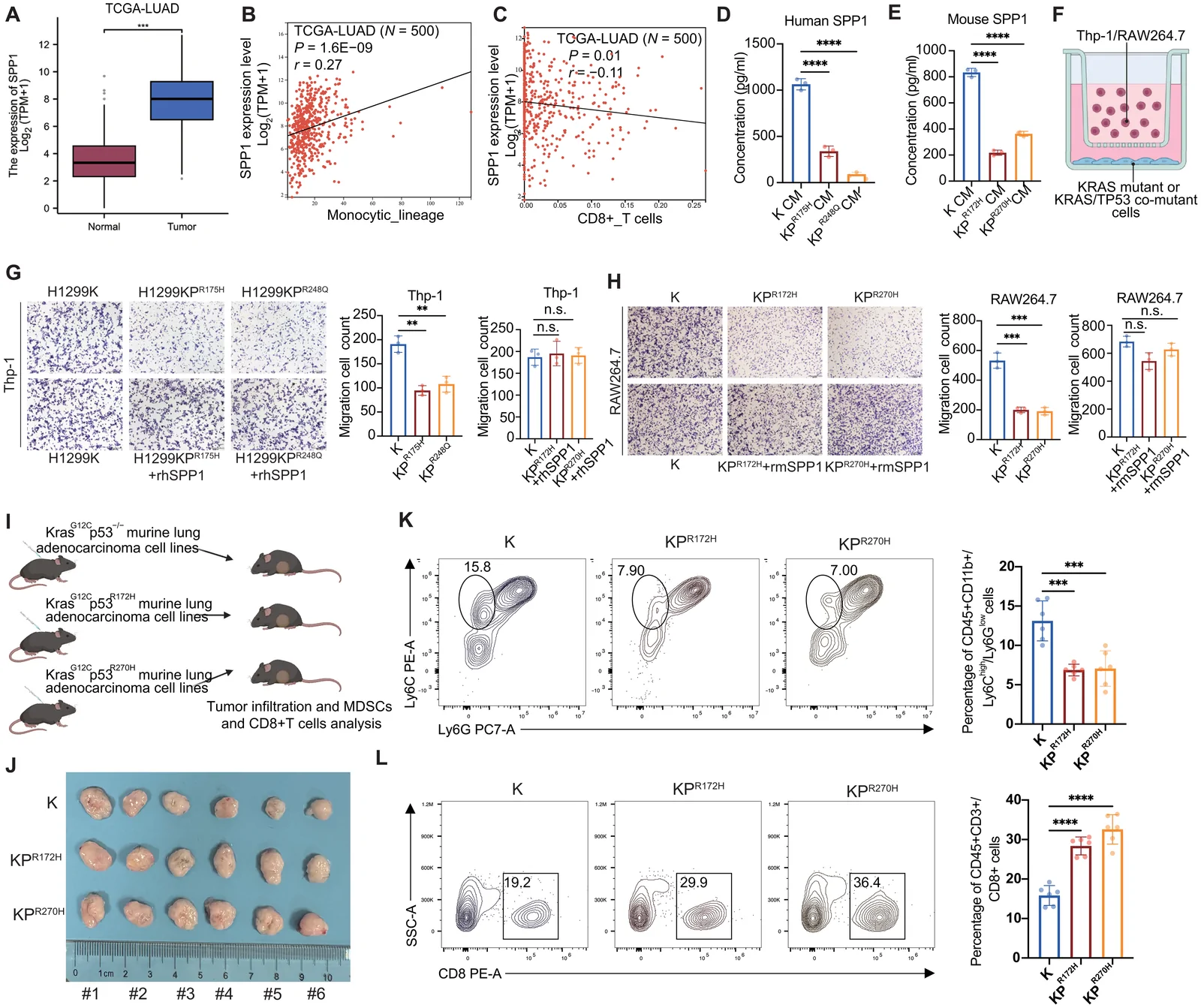
Mutant p53 impairs monocyte migration by reducing SPP1 secretion. (**A**) SPP1 mRNA expression level in tumor tissue versus adjacent normal tissue from the TCGA LUAD cohort. ***P < 0.001, two-tailed unpaired *t* test. (**B** and **C**) Infiltration abundance of monocyte (B) and CD8+ T cells (C) in TCGA-LUAD samples, deconvoluted using the xCell algorithm. Correlations were analyzed via Pearson’s correlation test. (**D**) ELISA quantification of SPP1 secretion in CM from H1299 cells harboring *KRAS* mutation (H1299K), KRAS/TP53^R175H^ (H1299KP^R175H^), and *KRAS/TP53*^R248Q^ (H1299KP^R248Q^). Four-parameter logistic (4PL) standard curve in fig. S3C. Data are means ± SD (*n* = 3). ****P < 0.0001, one-way ANOVA. (**E**) ELISA quantification of SPP1 secretion in CM from KP cells harboring *KRAS* mutation (K), *KRAS/TP53*^R172H^ (KP^R172H^), and *KRAS/TP53*^R270H^ (KP^R270H^). 4PL standard curve in fig. S3D. Data are means ± SD (*n* = 3). ****P < 0.0001, one-way ANOVA. (**F**) Schematic diagram of the Transwell coculture system. Created in BioRender. Zhang, Z. (2026) https://BioRender.com/amdy7ga. (**G**) Thp-1 migration induced by coculture with H1299K, H1299KP^R175H^, and H1299KP^R248Q^ cells or co-mutant H1299KP^R175H^/H1299KP^R248Q^ cells supplemented with recombinant human SPP1 (rhSPP1). Data are means ± SD (*n* = 3). **P < 0.01; n.s., not significant, one-way ANOVA. (**H**) RAW264.7 migration induced by coculture with KP, KP^R172H^, and KP^R270H^ cells or co-mutant KP^R172H^/KP^R270H^ cells supplemented with recombinant mouse SPP1 (rmSPP1). Data are means ± SD (*n* = 3). ***P < 0.001; n.s., not significant, one-way ANOVA. (**I**) Schematic diagram of the in vivo xenograft model. Created in BioRender. Zhang, Z. (2026) https://BioRender.com/8cfrdz5. (**J**) Size of xenograft tumors from KP, KP^R172H^, and KP^R270H^ cells. (**K** and **L**) Representative flow cytometry plots (left) and quantification (right) of tumor-infiltrating M-MDSC cells (K) and CD8+ T cells (L) [additional replicates, including representative images corresponding to those in (K) and (L), are provided in fig. S4, G and H]. Data are means ± SD (*n* = 6). *P < 0.05; ***P < 0.001; ****P < 0.0001, one-way ANOVA.

Recent findings indicated that SPP1 promotes the formation of an immunosuppressive TME (*25*–*27*). To characterize the association between SPP1 expression and immune cell subsets in NSCLC, we first analyzed TCGA-LUAD transcriptomic data and found that *SPP1* is highly expressed in LUAD tumor tissues and is associated with poor prognosis (Fig. 4A and fig. S4A). Next, we conducted xCell algorithm and found that *SPP1* expression was negatively correlated with CD8+ T cell infiltration and positively correlated with monocyte lineages (Fig. 4, B and C). As a secreted protein, SPP1 exerts chemokine-like functions that influence monocyte migration (*23*, *28*). To test whether mutant p53 remodels the *KRAS*-mutated NSCLC TME by suppressing SPP1 secretion, we collected conditioned medium (CM) from the human H1299 cell lines K, KP^R175H^, and KP^R248Q^ cells, as well as from mouse cell lines KP^−/−^, KP^R172HArg172His(R172H)^, and KP^R270HArg270His(R270H)^, and determined the levels of SPP1 by enzyme-linked immunosorbent assay (ELISA). Our results showed that SPP1 concentrations were reduced in CM from *KRAS/TP53* co-mutant cells compared to *KRAS*-mutant cells (Fig. 4, D and E, and fig. S4, B and C). We further performed the Transwell migration assay using THP-1 and RAW264.7 monocytes cocultured with tumor cells and found that *KRAS/TP53* co-mutant cells induced less monocyte migration than *KRAS*-mutant cells; this impairment was rescued by supplementation with either human or mouse recombinant SPP1 (Fig. 4, F to H). However, coculture assays using THP-1 and RAW264.7 with *KRAS*-mutant and *KRAS/TP53* co-mutant tumor cells revealed no statistically significant differences in IRF8 or SPP1 expression levels in the monocytes (fig. S4, D and E). We further examined whether tumors harboring *KRAS/TP53* co-mutations modulate the TME differently than those with *KRAS* mutation alone in vivo. C57BL/6 mice were subcutaneously inoculated with KP cell lines overexpressing p53-R172H or p53-R270H (Fig. 4I). Our finding demonstrated that tumor volume did not significantly differ among groups (Fig. 4J and fig. S4F), but flow cytometry analysis revealed increased CD8+ T cell infiltration and decreased M-MDSC infiltration in *KRAS/TP53* co-mutant tumors (Fig. 4, K to L, and fig. S4, G and H). Overall, these results indicate that mutant p53 reshapes the *KRAS*-mutant NSCLC immune microenvironment by inhibiting SPP1 secretion, thereby reducing M-MDSC chemotaxis and promoting CD8+ T cell infiltration.

### KRAS up-regulates SPP1 expression via the ERK-STAT1 pathway

**Fig. 5.**
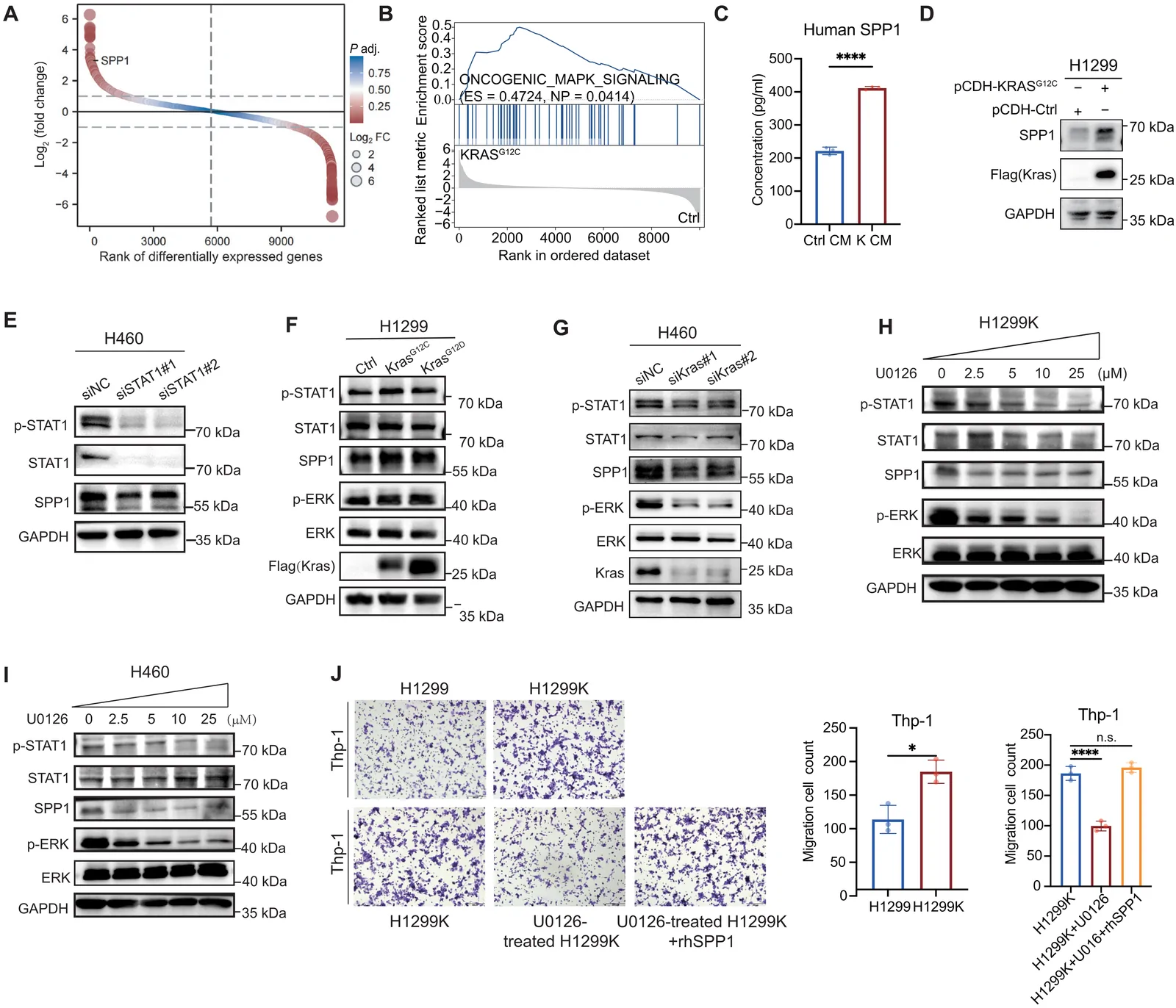
KRAS induces SPP1 expression via the ERK-STAT1 pathway. (**A**) Differential expression ranking plot of DEGs in *KRAS*-mutant versus wild-type H1299 cells. (**B**) GSEA plot showing the enrichment of MAPK signaling–related genes in *KRAS*-mutant cells. NES, normalized enrichment score; FDR, false discovery rate. (**C**) ELISA quantification of human SPP1 concentrations in CM from *KRAS* G12C mutant H1299 cells (H1299K) and H1299 cells (wild-type *KRAS*). Data are means ± SD (*n* = 3). ****P < 0.0001, two-tailed unpaired *t* test. (**D**) Western blot analysis of SPP1 and Flag-KRAS protein levels in H1299 cells transfected with pcDNA3.1-KRAS^G12C^ or pcDNA3.1-Ctrl. (**E**) Western blot analysis of p-STAT1, STAT1, and SPP1 protein levels in H460 cells transfected with siSTAT1 (#1/#2) or siNC. (**F**) Western blot analysis of p-STAT1, STAT1, SPP1, p-ERK, ERK, and Flag-KRAS protein levels in H1299 cells transfected with pcDNA3.1-KRAS^G12C^ or pcDNA3.1-Ctrl. (**G**) Western blot analysis of p-STAT1, STAT1, SPP1, p-ERK, ERK, and Kras protein levels in H460 cells transfected with siKRAS or siNC. (**H** and **I**) Western blot analysis of p-STAT1, STAT1, SPP1, p-ERK, and ERK protein levels in H1299K (H) and H460 (I) cells treated with increasing concentrations of U0126 (ERK inhibitor). (**J**) Thp-1 migration induced by coculture with H1299, H1299K cells, H1299K cells treated with U0126, or H1299K cells treated with U0126 and supplemented with recombinant human SPP1 (rhSPP1). Data are means ± SD (*n* = 3). *P < 0.05; ****P < 0.0001; n.s., not significant, one-way ANOVA.

We further investigated whether KRAS may mediate the expression of SPP1 expression. First, we analyzed transcriptomic data from H1299 cells with ectopic *KRAS* G12C expression. The results showed that SPP1 was up-regulated in the *KRAS*-mutant group, and gene set enrichment analysis (GSEA) (*29*) revealed that the MAPK pathway was enriched in this group (Fig. 5, A and B). We then validated these findings through cell-based experiments, which demonstrated that *KRAS* mutation not only up-regulates SPP1 expression at both the mRNA and protein levels but also enhances its secretion (Fig. 5, C and D, and fig. S3O). Because our results above identified *STAT1* as a transcriptional activator of SPP1 (Figs. 3C and 5E). In addition, a previous study had indicated that the MAPK-ERK signaling pathway can induce STAT1 phosphorylation (*30*). Therefore, we hypothesized that *KRAS* mutation may up-regulate SPP1 expression via the ERK-STAT1 signal axis. To this end, we first overexpressed mutant KRAS in H1299 cells and found showed that KRAS enhance ERK phosphorylation, which was accompanied by increased STAT1 phosphorylation and up-regulated SPP1 expression (Fig. 5F). Conversely, knockdown of endogenous mutant *KRAS* in H460 cells resulted in decreased ERK and STAT1 phosphorylation, as well as reduced SPP1 expression (Fig. 5G). We further used U0126, a selective inhibitor of ERK phosphorylation (*31*), to confirm the pathway dependency. H1299K cells and H460 cells were treated with U0126 at gradient concentrations, this treatment dose-dependently reduced ERK and STAT1 phosphorylation, as well as down-regulated SPP1 expression (Fig. 5, H and I). However, overexpression mutant p53 in H1299K cells not alter the phosphorylation levels of ERK or STAT1 (fig. S4I). Consistent with our findings, the Transwell migration assay demonstrated that H1299K cells induced increased migration of THP-1 cells compared with H1299 cells. H1299K cell–induced THP-1 migration was repressed by U0126 treatment, whereas this effect could be reversed by supplementation with rhSPP1 (Fig. 5J). Collectively, these results demonstrate that *KRAS* mutation drives ERK phosphorylation, which, in turn, leads to STAT1 phosphorylation and then the elevation of SPP1 expression and secretion, ultimately promoting monocyte migration.

### SPP1 blockade improves anti–PD-1 efficacy in *KRAS*-mutant LUAD

**Fig. 6.**
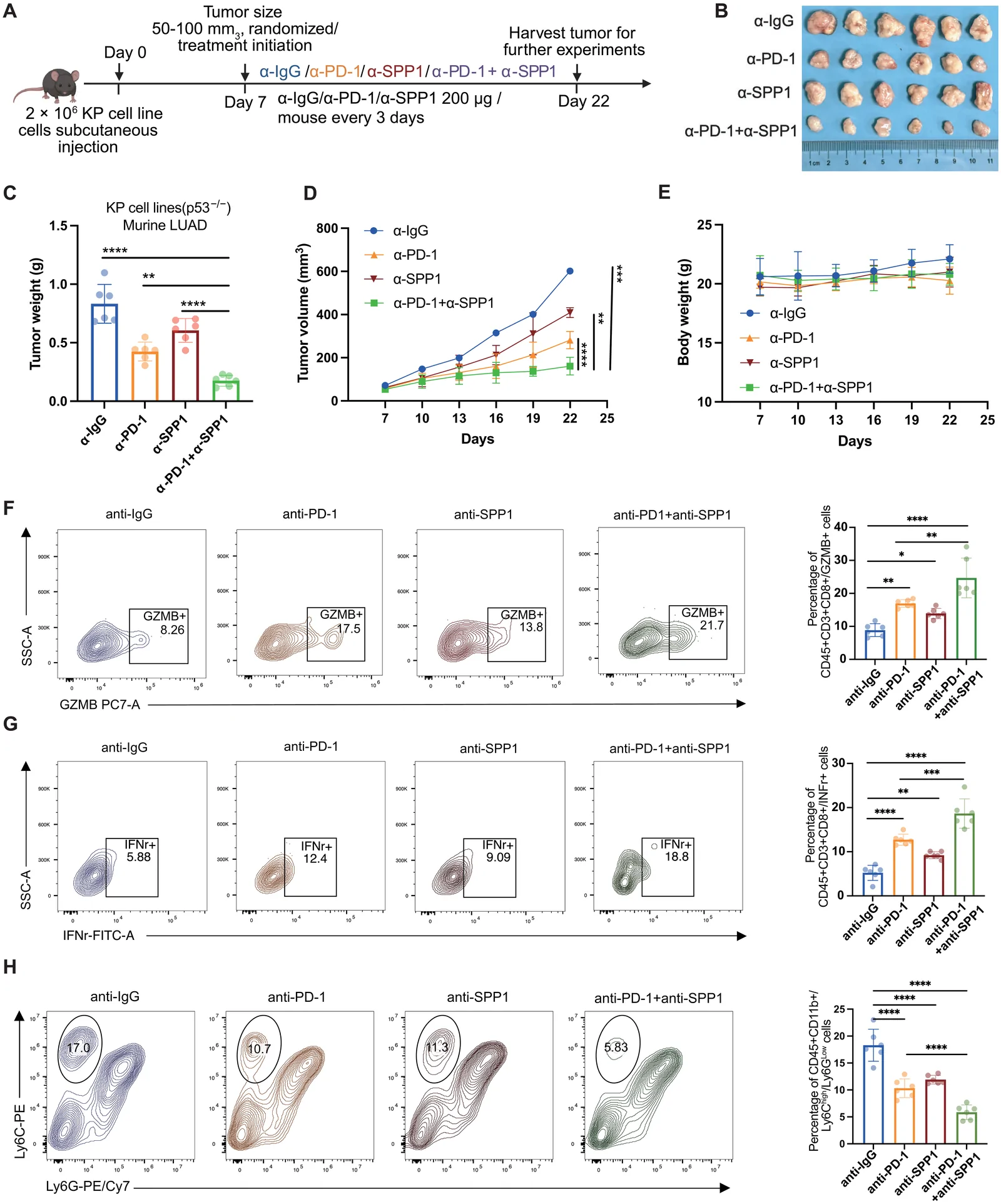
SPP1 blockade improves anti–PD-1 efficacy in KRAS-driven LUAD. (**A**) Schematic diagram of the in vivo therapeutic experiment. Created in BioRender. Zhang, Z. (2026) https://BioRender.com/8p64ru4. (**B** to **D**) Size (B), weight (C), and volume (D) of xenograft tumors from each treatment group. (**E**) Mouse body weight kinetics during the treatment period. Data are means ± SEM (*n* = 6). n.s., not significant. (**F** to **H**) Representative flow cytometry plots (left) and quantification (right) of tumor-infiltrating GZMB+CD8+ T cells (F), IFNγ+CD8+ T cells (G), and M-MDSC cells (H) [additional biological replicates, including representative images corresponding to those in (F) to (H), as well as full gating strategies, are provided in figs. S5, A and B, and S6, A to C] from each treatment group. Data are means ± SEM (*n* = 6). *P < 0.05; **P < 0.01; ***P < 0.001; ****P < 0.0001, one-way ANOVA.

Given that mutant p53 fosters an immunotherapy-favorable microenvironment by suppressing KRAS-induced SPP1, we hypothesized that SPP1 inhibition could enhance immunotherapy efficacy in KRAS-mutant LUAD in the absence of mutant p53. Accumulating preclinical evidence has validated the antitumor activity of SPP1-targeting antibodies in murine models (*22*, *32*, *33*). Thus, we sought to investigate whether anti-SPP1 could coordinate with anti–PD-1 to suppress *KRAS*-mutant LUAD growth in vivo. C57BL/6 mice were subcutaneously inoculated with KP cells (KRAS^G12D^/TP53^−/−^ mouse adenocarcinoma cell line) and randomly assigned to four groups, treated with anti–immunoglobulin G (IgG), anti–PD-1, anti-SPP1, or the combination of anti–PD-1 and anti-SPP1. All treatments were administered every 3 days for 2 weeks (Fig. 6A). Our results demonstrated that monotherapy with either agent reduced the tumor growth rate, volume, and weight relative to the control group (Fig. 6, B to D). The combination of anti-SPP1 and anti–PD-1 exerted a substantially greater antitumor effect than either monotherapy alone (Fig. 6, B to D). Notably, no significant body weight loss was observed across all treatment groups, indicating that the combination regimen was well tolerated in vivo (Fig. 6E). Flow cytometry analysis of tumor-infiltrating immune calls further revealed that combination therapy markedly increased the frequencies of GZMB+CD8+ cytotoxic T cells and IFN-γ+CD8+ effector T cells while concomitantly reducing the infiltration of M-MDSCs compared with the monotherapy groups (Fig. 6, F to H, and figs. S5, A and B, and S6, A to C). Collectively, our findings establish that the combination of anti-SPP1 and anti–PD-1 elicits synergistic antitumor immunity in *KRAS*-driven LUAD by remodeling the TME.

## DISCUSSION

**Fig. 7.**
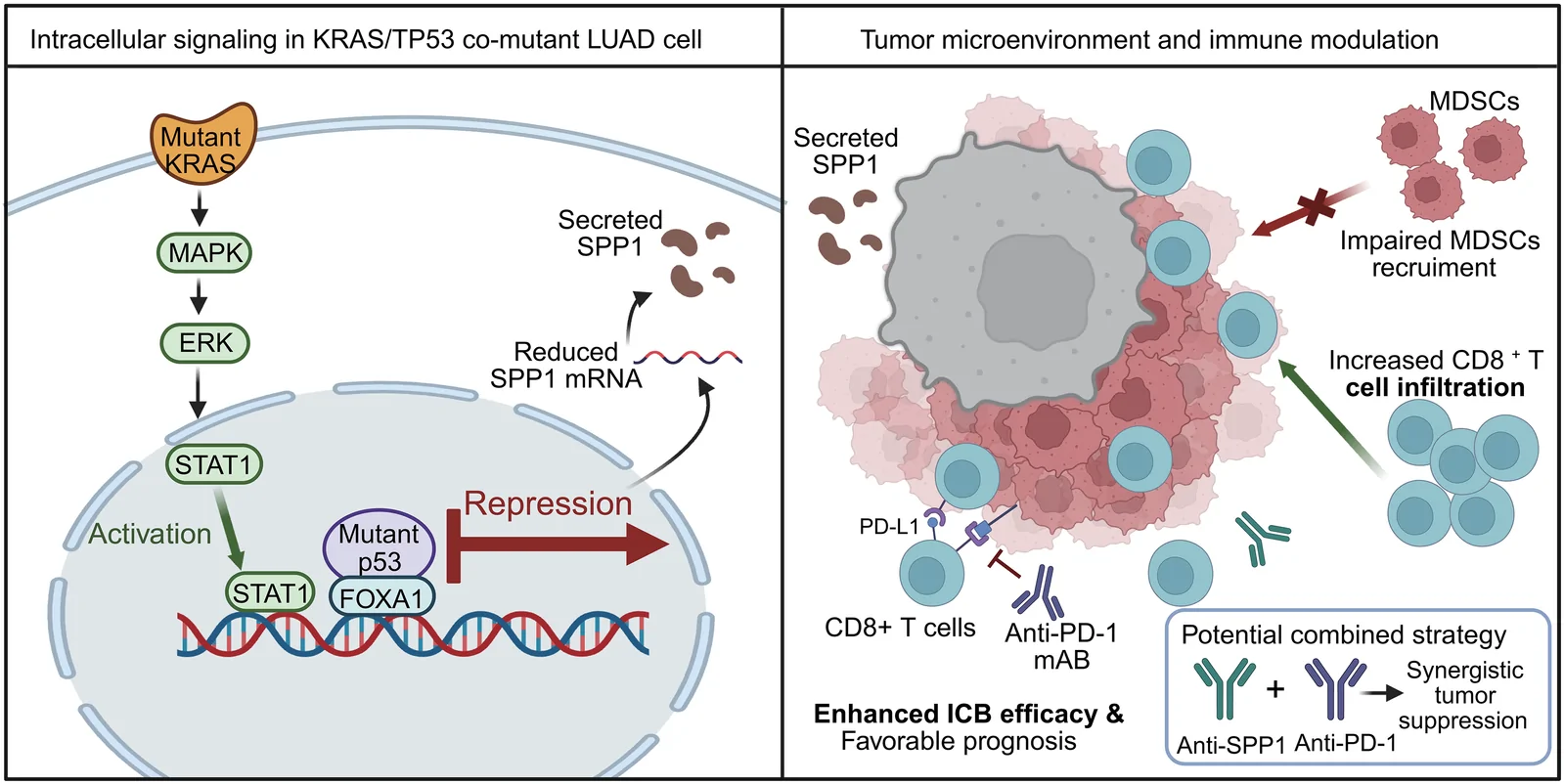
Schematic diagram of the study. Created in BioRender. Zhang, Z. (2026) https://BioRender.com/03dbtfi.

Although ICB has transformed the treatment landscape for advanced LUAD, therapeutic resistance remains a major clinical challenge (*34*, *35*). Accumulating evidence underscores that oncogene-defined LUAD subtypes exhibit considerable clinical heterogeneity in their response to immunotherapy, among which concurrent mutations with tumor suppressor genes emerging as a core determinant of this variability in oncogene-driven NSCLC subsets (*36*–*38*). Notably, for patients with *KRAS*-mutant NSCLC lacking access to first-line targeted therapies, co-mutation–based clinical subtyping is of particular significance for stratifying the efficacy of immunotherapy (*39*). Some study has classified *KRAS*-mutant LUAD into distinct subgroups, with the *KRAS/TP53* (KP) subtype exhibiting an inflamed TME characterized by enhanced CD8+ T cell infiltration and elevated PD-L1 expression (*19*, *40*, *41*). Our clinical data extend this observation by directly linking KP co-mutation to improved survival in ICB-treated patients (Fig. 1B), consistent with the Heidelberg cohort (HD-ICI) where patients with KRAS^MUT^TP53^MUT^ derived greater benefits from ICB than those with KRAS mutation alone (*18*). In addition, our study delineates a regulatory axis, mutant p53–FOXA1–SPP1, that modulates the TME to promote antitumor immunity (Fig. 7). These findings resolve the heterogeneous responses to ICB observed in *KRAS*-mutant LUAD and provide a rational, actionable strategy of combining SPP1 inhibition with anti–PD-1 therapy (Fig. 6, A to E, and fig. S7).

SPP1, an extracellular matrix glycoprotein rich in arginine–glycine–aspartic acid (RGD) motifs, binds to integrin receptors to mediate diverse functions (*20*, *42*, *43*). Accumulating evidence has established SPP1 as a pivotal regulator of TME remodeling, such as recruiting MDSCs and polarizing macrophages toward an immunosuppressive M2 phenotype, thereby establishing an immunosuppressive TME (*23*, *44*–*47*). Herein, we identify SPP1 as a convergent downstream target of *TP53* and *KRAS* mutations in LUAD, which are regulated in opposing ways: Mutant p53 cooperates with FOXA1 to repress SPP1 transcription, whereas oncogenic *KRAS* activates the MAPK-ERK-STAT1 axis to up-regulate SPP1 expression (Figs. 3, E to L, and 5, E to I). This dual regulatory model explains the heterogeneous SPP1 levels observed in *KRAS*-mutant LUAD. Specifically, in KP co-mutant tumors where mutant p53 predominates, SPP1 repression creates a favorable TME, which is characterized by reduced secretion, diminished M-MDSC recruitment, and enhanced CD8+ T cell infiltration, thereby providing a molecular basis for the improved immunotherapy response. Furthermore, the interaction between mutant p53 and FOXA1 adds a previously uncharacterized dimension to our understanding of mutant p53’s GOF properties. Although FOXA1 has been previously linked to LUAD progression, its role in immune regulation is poorly characterized (*48*–*50*). Our findings demonstrate that FOXA1 directly binds the SPP1 promoter to inhibit its transcription, and mutant p53 enhances this repression through protein-protein interaction with FOXA1. This mechanism expands the repertoire of mutant p53’s GOF activities in immune modulation. Concurrently, our study clarifies the pathway through which *KRAS* regulates SPP1. Constitutive MAPK activation by mutant *KRAS* induces ERK-dependent phosphorylation of STAT1, which subsequently transactivates the SPP1 promoter. This pathway links oncogenic *KRAS* signaling to TME remodeling, explaining why *KRAS* single-mutant tumors often exhibit high SPP1 levels and an immunosuppressive phenotype (Fig. 5J). Therefore, in KP co-mutant tumors, the SPP1-repressing effect of mutant p53 counteracts the SPP1-inducing activity of oncogenic *KRAS*. As a result, these tumors develop a more immune favorable TME than tumors with *KRAS* mutation alone.

The therapeutic implications of our findings are substantial, particularly in *KRAS*-driven LUAD. Given the key role of SPP1 in maintaining immunosuppression, we evaluated SPP1 blockade in combination with anti–PD-1 therapy in KRAS-mutant LUAD xenograft models (Fig. 6, A to E, and fig. S7). The synergistic antitumor effect observed in these models is biologically plausible. Specifically, anti-SPP1 reduces MDSC recruitment to relieve immunosuppression, whereas anti–PD-1 reinvigorates exhausted CD8+ T cells. This combination addresses two distinct layers of immune evasion, making it particularly effective in *KRAS*-mutant LUAD where SPP1-driven immunosuppression and PD-1–mediated T cell exhaustion coexist. Notably, the absence of significant body weight loss in treated mice further supports the safety of this combination, supporting its potential translation to clinical trials.

Although our study has yielded valuable insights into *KRAS/TP53* co-mutant LUAD, we also have several limitations that warrant consideration. First, although our clinical data validate the prognostic value of *KRAS/TP53* co-mutation, they are retrospective. Prospective studies with larger cohorts are needed to confirm the clinical utility of this biomarker and the efficacy of SPP1-targeted combination therapy. Second, although our study establishes the mutant p53–FOXA1–SPP1 axis as a key mediator of ICB sensitivity in *KRAS*-mutant LUAD through integrated clinical, cellular, and in vivo analyses, deeper mechanistic insight such as structural elucidation of the mutant p53–FOXA1–promoter complex and the link between the *TP53* and *KRAS* regulatory pathways would further strengthen the model. Third, although we focused on SPP1 as a key mediator, mutant p53 likely regulates other immune-related genes that contribute to ICB sensitivity. Future transcriptomic and proteomic profiling could uncover additional components of the mutant p53-driven immunoregulatory network. Last, although our study focuses on the mutant p53–FOXA1–SPP1 axis driven by hotspot missense mutations, we recognize that *TP53* mutation classes may have distinct biological consequences. Our analysis of the MSKCC immunotherapy cohort indicates that both missense and other loss-of-function (LOF) *TP53* mutations are associated with favorable clinical outcomes in *KRAS*-mutant LUAD. However, our mechanistic model specifically addresses GOF properties of some hotspot missense mutations. Whether LOF mutations elicit similar TME remodeling through alternative pathways or share common downstream targets remains to be elucidated. Future studies are required to comprehensively map the immunoregulatory networks governed by different classes of *TP53* alterations.

In conclusion, our study identifies SPP1 as a critical mediator of ICB efficacy in *KRAS*-mutant LUAD. Mechanistically, mutant p53 cooperates with FOXA1 to repress SPP1 transcription, whereas KRAS activates the MAPK-ERK-STAT1 pathway to up-regulate SPP1 expression. Our findings further indicate that, although oncogenic *KRAS* promotes an immunosuppressive TME by up-regulating SPP1, mutant p53 counteracts this effect to induce a more favorable TME, thereby enhancing response to immunotherapy. Last, our results demonstrate that SPP1 blockade can synergistically enhance anti–PD-1 therapy efficacy in KRAS-driven LUAD in the absence of mutant p53, providing a therapeutic strategy for this clinically challenging subset of lung cancer.

## MATERIALS AND METHODS

### Study cohorts and efficacy assessment

The FUSCC cohort consisted of 120 patients diagnosed with LUAD based on pathology from the FUSCC between July 2019 and August 2024. Inclusion criteria for patients included histologically confirmed diagnosis of stage III to IV LUAD, an absence of targetable EGFR/ALK/ROS1 mutations, with *KRAS* mutation or *KRAS/TP53* co-mutation, treatment-naïve status, and a baseline Eastern Cooperative Oncology Group (ECOG) performance status score of 0 or 1. Clinical stage was determined according to the eighth edition of the American Joint Committee on Cancer (AJCC) TNM staging system (*51*). All patients received ICIs in combination with chemotherapy every 3 weeks for four cycles, followed by ICI maintenance therapy every 3 weeks until disease progression or unacceptable toxicity occurred. PFS was defined as the time from the start of immunochemotherapy until either disease progression or death from any cause. OS was defined as the time from the start of immunochemotherapy until death from any cause. In addition to the FUSCC cohort, we also included data from the MSKCC cohort (*52*–*55*). The MSKCC cohort datasets are available on the cBioPortal website (www.cbioportal.org/) and 211 patients with LUAD receiving immunotherapy with *KRAS* mutation or *KRAS/TP53* co-mutation are included in our study.

### Cell culture and transient transfection

Mutant p53–harboring cancer cell lines, PC9, H1975, p53-null cell lines, H1299 and A549^p53−/−^, *KRAS* mutant cell lines H460, H1299K, and the murine immune cell line RAW264.7 and the human embryonic kidney cell line HEK293T were cultured in Dulbecco’s modified Eagle’s medium (DMEM). The p53-null cell line KP and the human immune cell line THP-1 were cultured in RPMI 1640 medium. Both media were supplemented with 10% fetal bovine serum (FBS), penicillin (100 U/ml), and streptomycin (0.1 mg/ml). All cells were maintained at 37°C in a humidified incubator with 5% CO_2_. All cell lines were regularly tested and confirmed free of mycoplasma contamination. For transient transfection, plasmids or siRNAs were delivered into cells using the Hieff Trans Liposomal Transfection Reagent (Yeasen, Shanghai, China) in accordance with the manufacturer’s protocol.

### Plasmids, antibodies, and reagents

The p53-encoding plasmids were described previously. Human *FOXA1* and *KRAS* were cloned into the vectors of Myc-pcDNA3.1 and Flag-pcDNA3.1, respectively. For the luciferase reporter assay, sequences upstream of the transcription initiation site of SPP1 were cloned into the vector pGL3-basic. The primers used for construction are listed in table S2. The anti-Flag (catalog no. F1805, Sigma-Aldrich, St Louis, MO, USA), anti-Myc (catalog no. 10828-1-AP, Proteintech), anti-p53 (catalog no. sc-126, DO-1, Santa Cruz Biotechnology), anti-SPP1 (catalog no. 22952-1-AP, Proteintech), anti-FOXA1 (catalog no. 20411-1-AP, Proteintech), anti-KRAS (catalog no. 12063-1-AP, Proteintech), anti-STAT1 (catalog no. T55227, Abmart), anti-Phospho-Stat1-Tyr701 (catalog no. 9167T, Cell Signaling Technology), anti-ERK (catalog no. 4695T, Cell Signaling Technology), anti-GAPDH (catalog no. 60004-1-Ig, Proteintech), and anti-Phospho-ERK-Thr202/Tyr204 (catalog no. 4370T, Cell Signaling Technology) were commercially purchased. The secondary antibodies used were horseradish peroxidase (HRP)-conjugated AffiniPure goat anti-rabbit IgG (catalog no.SA00001-2, Proteintech) and anti-mouse IgG (catalog no. SA00001-1, Proteintech). Proteins were visualized with the ECL chemiluminescence reagent (Yeasen). U0126 and the proteasome inhibitor were purchased from Sangon Biotech and MedChemExpress, respectively.

### Establishment of *KRAS*-mutant and *KRAS/TP53* co-mutant cell lines and RNA interference

We established our experimental models using two distinct genetic background cell lines: the human p53-null H1299 cell line and the murine KP cell line (derived from Kras^G12D^/Trp^null^ LUAD). For the human H1299 model, lentiviral particles were produced by cotransfecting HEK293T cells with the Flag-tagged KRAS^G12C^ plasmid and packaging plasmids psPAX2 and pMD2.G(5:3:2) using polyethylenimine (PEI; Sigma-Aldrich, USA). Viral supernatants were collected 48 hours posttransfection, filtered (0.45 μm), mixed 1:1 with fresh DMEM, and added to H1299 cells. At 24 hours postinfection, stable cells were selected using puromycin (2 μg/ml; MedChemExpress, Shanghai, China) for 48 hours. Expression of KRAS^G12C^ was validated by immunoblotting (IB). To generate *KRAS/TP53* co-mutant lines, these KRAS^G12C^-mutant H1299 cells were subsequently infected with lentiviruses carrying Flag-tagged control, Flag-tagged p53-R175H, or Flag-tagged p53-R248Q plasmids, following the same packaging and infection protocol. After infection, cells were selected with blasticidin (5 μg/ml) to obtain cells stably expressing both mutant transgenes. Coexpression of *KRAS* and *TP53* mutants was validated by IB. For the murine KP model, Flag-tagged p53-R172H or Flag-tagged p53-R270H plasmids were introduced via lentiviral transduction using the same protocol as described above. Transduced cells were selected using puromycin (2 μg/ml) to generate stable cell lines stably expressing respective p53 mutants. Confirmation of transgene expression was verified by IB. The siRNAs used in this study were synthesized and purified by GenePharma (Shanghai, China), and sequences are listed in table S3. siRNAs were introduced into cells using the Hieff Trans Liposomal transfection reagent following the manufacturer’s protocol (Yeasen). Cells were harvested 48 to 72 hours after transfection for IB or RT-qPCR.

### RNA sequencing and bioinformatic analysis

Total RNA was extracted from H1299 cells, *KRAS*-mutant cells, and *KRAS/TP53* co-mutant H1299 cells using RNAiso Plus (Takara, Japan) according to the manufacturer’s protocol. RNA sequencing (RNA-seq) and subsequent bioinformatic analysis was performed by OE Biotech (Shanghai, China). Differentially expressed mRNAs were identified using the limma package in the R (*56*), with thresholds set at an adjusted *P* value of <0.05 and an absolute log_2_(FC) of > 1. The ggplot2 package in the R software was used to draw the volcano plot. Functional annotation and pathway enrichment analyses were conducted using the ClusterProfiler package (version: 3.18.0) for Gene Ontology (GO) and KEGG pathways (*57*).

### Reverse transcription and quantitative real-time PCR

Total RNA was extracted from cells using RNAiso Plus (Takara, Japan) according to the manufacturer’s protocol. Genomic DNA (gDNA) was extracted from cells using the TIANamp Genomic DNA kit (TIANGEN, China) according to the provided protocol. cDNA was synthesized from the extracted RNA using the HiScriptIII RT SuperMix for qPCR (+gDNA wiper) (Vazyme, China). RT-qPCR analysis was performed using the SYBR qPCR Master Mix as directed (Vazyme, China). The relative expression levels of target genes were calculated using the comparative ΔΔCt method. *GAPDH* (glyceraldehyde-3-phosphate dehydrogenase) was used as an internal control. The primers for RT-qPCR are listed in table S4.

### Immunoblotting

Proteins were extracted using radioimmunoprecipitation assay (RIPA) buffer. The extracts were separated by SDS-polyacrylamide gel electrophoresis (PAGE) and subsequently transferred onto polyvinylidene difluoride membranes. After blocking with 5% nonfat milk for 1 hour at room temperature, the membranes were incubated with primary antibodies overnight at 4°C. Following three washes with TBST, the membranes were incubated with HRP-conjugated secondary antibodies diluted at 1:10,000 for 1 hour at room temperature. Proteins were visualized using the ECL chemiluminescence reagent (Yeasen, China) and imaged with a chemiluminescence detection system.

### Determine the secretion of endogenous SPP1

To determine the endogenous SPP1 secretion, *KRAS*-mutant cells and *KRAS/TP53* co-mutant cells were seeded in 10-cm dishes and cultured under standard conditions until they reached 80% confluence. The culture medium was then replaced with serum-free conditioned medium (SFCM) for 48 hours. Subsequently, the supernatant was collected and filtered through a 0.45-μm filter. Levels of secreted SPP1 in SFCM were quantified using ELISA kits (catalog no. KE10118, KE00233, Proteintech, China) according to the manufacturer’s protocols.

### In vitro coculture with tumor cell and RAW264.7 or THP-1

Coculture assays were performed using Transwell chambers to evaluate the recruitment of RAW264.7 or THP-1 cells. Briefly, 1 × 10^5^ *KRAS*-mutant or *KRAS/TP53* co-mutant cells were plated in the lower chamber of a 24-well plate in 800 μL of culture medium containing 20% FBS. Then, 5 × 10^4^ RAW264.7 or PMA-treated (catalog no. 50601ES03, Yeasen, China) THP-1 cells were seeded into the upper chambers (pore size, 8 μm; Corning, NY, USA). After incubation at 37°C for 36–48 hours, nonmigratory cells on the upper surface of the membrane were gently removed with a cotton swab. Cells that migrated to the lower surface were fixed with methanol and stained with 0.2% crystal violet. The number of migrated cells was quantified by counting at least three randomly selected fields under an optical microscope and analyzed using ImageJ.

### IP assay

For exogenous co-IP, HEK293T cells were transfected with the indicated plasmids using PEI for 36 to 48 hours and treated with MG132 for 4 to 6 hours before being harvested. For endogenous IP, PC9 cells were transfected with Flag-pcDNA3.1-FOXA1 for 36 to 48 hours and treated with MG132 for 4 to 6 hours before being harvested. The IP assay was conducted using antibodies as indicated in the figure legends. Briefly, 500 to 1000 μg of protein lysates was incubated with specified antibody at 4°C for 5 hours. Protein A or G beads (Santa Cruz Biotechnology) were then added, and the mixture was incubated for additional 2 hours at 4°C. The beads were washed six to eight times with lysis buffer. Protein interactions were detected by IB as described above.

### Luciferase reporter assay

Cells were seeded in 24-well plates and cotransfected with pGL3-basic, pGL3-SPP1, or pGL3-SPP1-DEL1/2/3 plasmids, along with plasmids encoding Renilla luciferase and *FOXA1*. After 48 hours of culture, the cells were harvested and luciferase activity was measured using a Dual-Luciferase Reporter Assay System (Vazyme, China) according to the manufacturer’s protocol.

### Chromatin immunoprecipitation

Cells were seeded in 10-cm dishes and cultured for 24 hours. Cross-linking was performed using 37% formaldehyde for 10 min at room temperature, neutralized with 800 μl of 10× glycine for 5 min, and washed three times with cold phosphate-buffered saline (PBS). Cells were scraped and resuspended in cell lysis buffer [50 mM tris-HCl (pH 7.5), 140 mM NaCl, 1 mM EDTA, 10% glycerol, 0.5% NP-40, 0.25% Triton X-100, and protease inhibitor cocktail] and incubated on ice for 20 min with vortexing every 5 min. Nuclei were collected and resuspended in 400 μL nuclear lysis buffer [50 mM tris-HCl (pH 8.0), 10 mM EDTA, 1% SDS, and protease inhibitor cocktail]. After sonication (200 W, 60 cycles with 30-s on and 30-s off) and centrifuged at 12,000*g* for 5 min at 4°C, and the supernatants were mixed with anti-p53 or IgG at 4°C overnight and then with protein A/G beads at 4°C for 2 hours. Beads were washed sequentially with Low Salt Wash Buffer [50 mM tris-HCl (pH 8.0), 0.1% SDS, 0.5% deoxycholate, 1 mM EDTA, 1% NP-40, and 150 mM NaCl], High Salt Wash Buffer [50 mM tris-HCl (pH 8.0), 0.1% SDS, 0.5% deoxycholate, 1 mM EDTA, 1% NP-40, and 500 mM NaCl], LiCl Wash Buffer [50 mM tris-HCl (pH 8.0), 250 mM LiCl, 0.1% SDS, 0.5% deoxycholate, 1 mM EDTA, and 1% NP-40], and TE Buffer [10 mM tris-HCl (pH 8.0) and 1 mM EDTA]. Protein-DNA complexes were eluted using ChIP elution buffer (1% SDS and 0.1 M NaHCO_3_). After decross-linking at 62°C for 2 hours, DNA was purified and analyzed by qPCR and agarose gel electrophoresis. The primers used are as follows: ChIP-F: (5′-3′); ChIP-R: (5′-3′).

### Mouse xenograft study

Five-week-old female C57BL/6 mice were purchased from and housed in the Laboratory Animal Science of Fudan University Shanghai Cancer Center. To establish orthotopic tumor xenograft models, 2 × 10^6^ KP cells or KP cells stably expressing mutant p53–R172H or p53-R270H suspended in RPMI 1640 medium were injected into the right flanks of the mice. Tumor growth was monitored using an electronic digital caliper by measuring in two dimensions. Tumor volume was calculated according to the formula: tumor volume (mm^3^) = (length × width^2^) × 0.52. For drug treatment, the mice were randomized assigned to treatment groups after the tumor volume reached 50 to 100 mm^3^. Each mouse received intraperitoneal injections of 200 μg of anti-IgG, anti–PD-1, anti-SPP1, or anti-SPP1 plus anti–PD-1 antibody (BioXcell, USA) every 3 days, and the tumor size was measured after each administration of the therapeutic agents. Last, the mice were euthanized, and tumors were collected for analysis. All animal procedures were conducted in compliance with ethical guidelines and were approved by the Animal Welfare Committee of FUSCC.

### Flow cytometry assay

Tumor tissues were collected from mice and digested into single-cell suspensions using the Gentle Enzymatic Digestion Kit for Mouse Tumor Tissue (RWD, China) according to the manufacturer’s protocol. Cell suspension was sequentially filtered through 100- and 40-μm cell strainers. Following erythrocytes lysis, the cells were incubated with fluorochrome-conjugated antibodies (table S4) for 30 min at 4°C in the dark. Data were acquired on a flow cytometer and analyzed using the CytExpert software (Beckman Coulter).

### Statistical analysis

All in vitro data were obtained from three independent replicate experiments. Data were shown as means ± SD. A two-tailed unpaired *t* test or one-way analysis of variance (ANOVA) was used to compare the differences between two or more groups. The PFS and OS of patients were analyzed using the Kaplan-Meier method. *P* < 0.05 was considered statistically significant. ns, not significant; *P < 0.05, **P < 0.01, ***P < 0.001, and ****P < 0.0001.

### Ethics statements

The study was approved by the Institutional Review Board of the Fudan University Shanghai Cancer Center (approval no. 2004216-20-2005) and was performed in compliance with the Helsinki declaration. The animal study was approved by the ethics committee of the Fudan University Shanghai Cancer Center (grant no. FUSCC-IACUC-2024492).

## References

[1] R. L. Siegel, T. B. Kratzer, A. N. Giaquinto, H. Sung, A. Jemal, Cancer statistics, 2025. CA Cancer J. Clin. 75, 10–45 (2025). 39817679 10.3322/caac.21871 PMC11745215

[2] D. Rodríguez-Abreu, S. F. Powell, M. J. Hochmair, S. Gadgeel, E. Esteban, E. Felip, G. Speranza, F. De Angelis, M. Dómine, S. Y. Cheng, H. G. Bischoff, N. Peled, M. Reck, R. Hui, E. B. Garon, M. Boyer, T. Kurata, J. Yang, M. C. Pietanza, F. Souza, M. C. Garassino, Pemetrexed plus platinum with or without pembrolizumab in patients with previously untreated metastatic nonsquamous NSCLC: Protocol-specified final analysis from KEYNOTE-189. Ann. Oncol. 32, 881–895 (2021). 33894335 10.1016/j.annonc.2021.04.008

[3] T. S. K. Mok, Y. L. Wu, I. Kudaba, D. M. Kowalski, B. C. Cho, H. Z. Turna, G. Castro Jr., V. Srimuninnimit, K. K. Laktionov, I. Bondarenko, K. Kubota, G. M. Lubiniecki, J. Zhang, D. Kush, G. Lopes, KEYNOTE-042 Investigators, Pembrolizumab versus chemotherapy for previously untreated, PD-L1-expressing, locally advanced or metastatic non-small-cell lung cancer (KEYNOTE-042): A randomised, open-label, controlled, phase 3 trial. Lancet 393, 1819–1830 (2019). 30955977 10.1016/S0140-6736(18)32409-7

[4] M. C. Garassino, S. Gadgeel, G. Speranza, E. Felip, E. Esteban, M. Dómine, M. J. Hochmair, S. F. Powell, H. G. Bischoff, N. Peled, F. Grossi, R. R. Jennens, M. Reck, R. Hui, E. B. Garon, T. Kurata, J. E. Gray, P. Schwarzenberger, E. Jensen, M. C. Pietanza, D. Rodríguez-Abreu, Pembrolizumab plus pemetrexed and platinum in nonsquamous non-small-cell lung cancer: 5-year outcomes from the phase 3 KEYNOTE-189 study. J. Clin. Oncol. 41, 1992–1998 (2023). 36809080 10.1200/JCO.22.01989 PMC10082311

[5] M. D. Vesely, T. Zhang, L. Chen, Resistance mechanisms to anti-PD cancer immunotherapy. Annu. Rev. Immunol. 40, 45–74 (2022). 35471840 10.1146/annurev-immunol-070621-030155

[6] A. J. Schoenfeld, M. D. Hellmann, Acquired resistance to immune checkpoint inhibitors. Cancer Cell 37, 443–455 (2020). 32289269 10.1016/j.ccell.2020.03.017 PMC7182070

[7] D. Memon, A. J. Schoenfeld, D. Ye, G. Fromm, H. Rizvi, X. Zhang, M. R. Keddar, D. Mathew, K. J. Yoo, J. Qiu, J. Lihm, J. Miriyala, J. L. Sauter, J. Luo, A. Chow, U. K. Bhanot, C. McCarthy, C. M. Vanderbilt, C. Liu, M. Abu-Akeel, A. J. Plodkowski, N. McGranahan, M. Łuksza, B. D. Greenbaum, T. Merghoub, I. Achour, J. C. Barrett, R. Stewart, P. Beltrao, T. H. Schreiber, A. J. Minn, M. L. Miller, M. D. Hellmann, Clinical and molecular features of acquired resistance to immunotherapy in non-small cell lung cancer. Cancer Cell 42, 209–224.e9 (2024). 38215748 10.1016/j.ccell.2023.12.013 PMC11249385

[8] M. Reck, D. P. Carbone, M. Garassino, F. Barlesi, Targeting KRAS in non-small-cell lung cancer: Recent progress and new approaches. Ann. Oncol. 32, 1101–1110 (2021). 34089836 10.1016/j.annonc.2021.06.001

[9] F. Barlesi, W. Yao, M. Duruisseaux, L. Doucet, A. A. Martínez, V. Gregorc, O. Juan-Vidal, S. Lu, C. De Bondt, F. de Marinis, H. Linardou, Y. C. Kim, R. Jotte, E. Felip, G. Lo Russo, M. Reck, M. F. Michenzie, W. Yang, J. N. Meade, B. Korytowsky, T. S. K. Mok, KRYSTAL-12 Investigators, Adagrasib versus docetaxel in *KRAS*^G12C^-mutated non-small-cell lung cancer (KRYSTAL-12): A randomised, open-label, phase 3 trial. Lancet 406, 615–626 (2025). 40783289 10.1016/S0140-6736(25)00866-9

[10] R. A. S. Drugging, Drugging RAS: Moving beyond KRAS^G12C^. Cancer Discov. 13, OF7 (2023). 10.1158/2159-8290.CD-ND2023-0010 37871268

[11] Z. Chen, K. Cheng, Z. Walton, Y. Wang, H. Ebi, T. Shimamura, Y. Liu, T. Tupper, J. Ouyang, J. Li, P. Gao, M. S. Woo, C. Xu, M. Yanagita, A. Altabef, S. Wang, C. Lee, Y. Nakada, C. G. Peña, Y. Sun, Y. Franchetti, C. Yao, A. Saur, M. D. Cameron, M. Nishino, D. N. Hayes, M. D. Wilkerson, P. J. Roberts, C. B. Lee, N. Bardeesy, M. Butaney, L. R. Chirieac, D. B. Costa, D. Jackman, N. E. Sharpless, D. H. Castrillon, G. D. Demetri, P. A. Jänne, P. P. Pandolfi, L. C. Cantley, A. L. Kung, J. A. Engelman, K. K. Wong, A murine lung cancer co-clinical trial identifies genetic modifiers of therapeutic response. Nature 483, 613–617 (2012). 22425996 10.1038/nature10937 PMC3385933

[12] B. Ricciuti, K. C. Arbour, J. J. Lin, A. Vajdi, N. Vokes, L. Hong, J. Zhang, M. Y. Tolstorukov, Y. Y. Li, L. F. Spurr, A. D. Cherniack, G. Recondo, G. Lamberti, X. Wang, D. Venkatraman, J. V. Alessi, V. R. Vaz, H. Rizvi, J. Egger, A. J. Plodkowski, S. Khosrowjerdi, S. Digumarthy, H. Park, N. Vaz, M. Nishino, L. M. Sholl, D. Barbie, M. Altan, J. V. Heymach, F. Skoulidis, J. F. Gainor, M. D. Hellmann, M. M. Awad, Diminished efficacy of programmed death-(ligand) 1 inhibition in STK11- and KEAP1-mutant lung adenocarcinoma is affected by KRAS mutation status. J. Thorac. Oncol. 17, 399–410 (2022). 34740862 10.1016/j.jtho.2021.10.013 PMC10980559

[13] Z. Y. Dong, W. Z. Zhong, X. C. Zhang, J. Su, Z. Xie, S. Y. Liu, H. Y. Tu, H. J. Chen, Y. L. Sun, Q. Zhou, J. J. Yang, X. N. Yang, J. X. Lin, H. H. Yan, H. R. Zhai, L. X. Yan, R. Q. Liao, S. P. Wu, Y. L. Wu, Potential predictive value of TP53 and KRAS mutation status for response to PD-1 blockade immunotherapy in lung adenocarcinoma. Clin. Cancer Res. 23, 3012–3024 (2017). 28039262 10.1158/1078-0432.CCR-16-2554

[14] Q. Hao, J. Chen, H. Lu, X. Zhou, The ARTS of p53-dependent mitochondrial apoptosis. J. Mol. Cell Biol. 14, mjac074 (2023). 36565718 10.1093/jmcb/mjac074 PMC10053023

[15] X. Zhou, Q. Hao, H. Lu, Mutant p53 in cancer therapy—The barrier or the path. J. Mol. Cell Biol. 11, 293–305 (2019). 30508182 10.1093/jmcb/mjy072 PMC6487791

[16] M. Ghosh, S. Saha, J. Bettke, R. Nagar, A. Parrales, T. Iwakuma, A. W. M. van der Velden, L. A. Martinez, Mutant p53 suppresses innate immune signaling to promote tumorigenesis. Cancer Cell 39, 494–508.e5 (2021). 33545063 10.1016/j.ccell.2021.01.003 PMC8044023

[17] M. Gu, T. Xu, P. Chang, *KRAS*/*LKB1* and *KRAS*/*TP53* co-mutations create divergent immune signatures in lung adenocarcinomas. Ther. Adv. Med. Oncol. 13, 17588359211006950 (2021). 33995590 10.1177/17588359211006950 PMC8072935

[18] J. Budczies, E. Romanovsky, M. Kirchner, O. Neumann, M. Blasi, J. Schnorbach, R. Shah, F. Bozorgmehr, R. Savai, T. Stiewe, S. Peters, P. Schirmacher, M. Thomas, D. Kazdal, P. Christopoulos, A. Stenzinger, KRAS and TP53 co-mutation predicts benefit of immune checkpoint blockade in lung adenocarcinoma. Br. J. Cancer 131, 524–533 (2024). 38866964 10.1038/s41416-024-02746-z PMC11300455

[19] S. B. Urtecho, L. Provenzano, A. Spagnoletti, A. Bottiglieri, C. Pircher, G. Massa, C. Sposetti, C. Proto, M. Brambilla, M. Occhipinti, L. Mazzeo, T. Beninato, R. Leporati, C. Giani, C. Cavalli, R. Serino, M. M. Prina, A. Bassetti, V. Nasca, R. M. di Mauro, A. Abate, S. Manglaviti, A. D. Dumitrascu, G. D. Liberti, T. S. Cassano, M. Ganzinelli, S. Wu, M. C. Garassino, F. G. M. de Braud, G. L. Russo, A. Prelaj, Decoding KRAS mutation in non-small cell lung cancer patients receiving immunotherapy: A retrospective institutional comparison and literature review. Lung Cancer 199, 108051 (2025). 39740426 10.1016/j.lungcan.2024.108051

[20] H. Zhao, Q. Chen, A. Alam, J. Cui, K. C. Suen, A. P. Soo, S. Eguchi, J. Gu, D. Ma, The role of osteopontin in the progression of solid organ tumour. Cell Death Dis. 9, 356 (2018). 29500465 10.1038/s41419-018-0391-6 PMC5834520

[21] L. Liu, R. Zhang, J. Deng, X. Dai, X. Zhu, Q. Fu, H. Zhang, Z. Tong, P. Zhao, W. Fang, Y. Zheng, X. Bao, Construction of TME and Identification of crosstalk between malignant cells and macrophages by SPP1 in hepatocellular carcinoma. Cancer Immunol. Immunother. 71, 121–136 (2022). 34028567 10.1007/s00262-021-02967-8 PMC10992184

[22] Y. Liu, Z. Xun, K. Ma, S. Liang, X. Li, S. Zhou, L. Sun, Y. Liu, Y. Du, X. Guo, T. Cui, H. Zhou, J. Wang, D. Yin, R. Song, S. Zhang, W. Cai, F. Meng, H. Guo, B. Zhang, D. Yang, R. Bao, Q. Hu, J. Wang, Y. Ye, L. Liu, Identification of a tumour immune barrier in the HCC microenvironment that determines the efficacy of immunotherapy. J. Hepatol. 78, 770–782 (2023). 36708811 10.1016/j.jhep.2023.01.011

[23] D. Brina, A. Ponzoni, M. Troiani, B. Calì, E. Pasquini, G. Attanasio, S. Mosole, M. Mirenda, M. D’Ambrosio, M. Colucci, I. Guccini, A. Revandkar, A. Alajati, T. Tebaldi, D. Donzel, F. Lauria, N. Parhizgari, A. Valdata, M. Maddalena, A. Calcinotto, M. Bolis, A. Rinaldi, S. Barry, J. H. Rüschoff, M. Sabbadin, S. Sumanasuriya, M. Crespo, A. Sharp, W. Yuan, M. Grinu, A. Boyle, C. Miller, L. Trotman, N. Delaleu, M. Fassan, H. Moch, G. Viero, J. de Bono, A. Alimonti, The Akt/mTOR and MNK/eIF4E pathways rewire the prostate cancer translatome to secrete HGF, SPP1 and BGN and recruit suppressive myeloid cells. Nat. Cancer 4, 1102–1121 (2023). 37460872 10.1038/s43018-023-00594-z PMC11331482

[24] L. J. Padrón, D. M. Maurer, M. H. O’Hara, E. M. O’Reilly, R. A. Wolff, Z. A. Wainberg, A. H. Ko, G. Fisher, O. Rahma, J. P. Lyman, C. R. Cabanski, J. X. Yu, S. M. Pfeiffer, M. Spasic, J. Xu, P. F. Gherardini, J. Karakunnel, R. Mick, C. Alanio, K. T. Byrne, T. J. Hollmann, J. S. Moore, D. D. Jones, M. Tognetti, R. O. Chen, X. Yang, L. Salvador, E. J. Wherry, U. Dugan, J. O’Donnell-Tormey, L. H. Butterfield, V. M. Hubbard-Lucey, R. Ibrahim, J. Fairchild, S. Bucktrout, T. M. LaVallee, R. H. Vonderheide, Sotigalimab and/or nivolumab with chemotherapy in first-line metastatic pancreatic cancer: Clinical and immunologic analyses from the randomized phase 2 PRINCE trial. Nat. Med. 28, 1167–1177 (2022). 35662283 10.1038/s41591-022-01829-9 PMC9205784

[25] H. Zheng, Y.-Q. Li, X. Lu, J. Zhang, S.-S. Yu, X.-F. Deng, X.-B. Liu, M.-Y. Li, Y. Cao, Q. Chen, Y. Qiu, Q.-X. Liu, D. Zhou, J. G. Dai, Senescent SPP1^+^ macrophages remodel the tumor microenvironment and promote the progression of early-stage lung adenocarcinoma featured with mixed ground glass nodule. Mol. Cancer 24, 298 (2025). 41310768 10.1186/s12943-025-02497-2 PMC12659132

[26] J. Shi, S. Huang, Y. Zhou, G. Zheng, Z. Zhang, X. Fan, J. Shen, SPP1^+^ neutrophils mediate resistance to immune checkpoint blockade in BAP1-inactivated tumors. Cancer Res. 85, 4433–4449 (2025). 40939189 10.1158/0008-5472.CAN-24-4962

[27] R. Trehan, P. Huang, X. B. Zhu, X. Wang, M. Soliman, D. Strepay, A. Nur, N. Kedei, M. Arhin, S. Ghabra, F. Rodríguez-Matos, M. R. Benmebarek, C. Ma, F. Korangy, T. F. Greten, SPP1+ macrophages cause exhaustion of tumor-specific T cells in liver metastases. Nat. Commun. 16, 4242 (2025). 40335453 10.1038/s41467-025-59529-0 PMC12059142

[28] M. R. Shurin, Osteopontin controls immunosuppression in the tumor microenvironment. J. Clin. Invest. 128, 5209–5212 (2018). 30395537 10.1172/JCI124918 PMC6264653

[29] A. Subramanian, P. Tamayo, V. K. Mootha, S. Mukherjee, B. L. Ebert, M. A. Gillette, A. Paulovich, S. L. Pomeroy, T. R. Golub, E. S. Lander, J. P. Mesirov, Gene set enrichment analysis: A knowledge-based approach for interpreting genome-wide expression profiles. Proc. Natl. Acad. Sci. U.S.A. 102, 15545–15550 (2005). 16199517 10.1073/pnas.0506580102 PMC1239896

[30] B. Cui, J. Chen, M. Luo, Y. Liu, H. Chen, D. Lü, L. Wang, Y. Kang, Y. Feng, L. Huang, P. Zhang, PKD3 promotes metastasis and growth of oral squamous cell carcinoma through positive feedback regulation with PD-L1 and activation of ERK-STAT1/3-EMT signalling. Int. J. Oral Sci. 13, 8 (2021). 33692335 10.1038/s41368-021-00112-w PMC7946959

[31] S. Nishimoto, E. Nishida, MAPK signalling: ERK5 versus ERK1/2. EMBO Rep. 7, 782–786 (2006). 16880823 10.1038/sj.embor.7400755 PMC1525153

[32] H. Wang, N. Li, Q. Liu, J. Guo, Q. Pan, B. Cheng, J. Xu, B. Dong, G. Yang, B. Yang, X. Wang, Y. Gu, G. Zhang, Y. Lian, W. Zhang, M. Zhang, T. Li, Y. Zang, M. Tan, Q. Li, X. Wang, Z. Yu, J. Jiang, H. Huang, J. Qin, Antiandrogen treatment induces stromal cell reprogramming to promote castration resistance in prostate cancer. Cancer Cell 41, 1345–1362.e9 (2023). 37352863 10.1016/j.ccell.2023.05.016

[33] H. He, S. Chen, Z. Fan, Y. Dong, Y. Wang, S. Li, X. Sun, Y. Song, J. Yang, Q. Cao, J. Jiang, X. Wang, W. Wen, H. Wang, Multi-dimensional single-cell characterization revealed suppressive immune microenvironment in AFP-positive hepatocellular carcinoma. Cell Discov. 9, 60 (2023). 37336873 10.1038/s41421-023-00563-x PMC10279759

[34] M. L. Meyer, B. G. Fitzgerald, L. Paz-Ares, F. Cappuzzo, P. A. Jänne, S. Peters, F. R. Hirsch, New promises and challenges in the treatment of advanced non-small-cell lung cancer. Lancet 404, 803–822 (2024). 39121882 10.1016/S0140-6736(24)01029-8

[35] A. Passaro, J. Brahmer, S. Antonia, T. Mok, S. Peters, Managing resistance to immune checkpoint inhibitors in lung cancer: Treatment and novel strategies. J. Clin. Oncol. 40, 598–610 (2022). 34985992 10.1200/JCO.21.01845

[36] A. Ravi, M. D. Hellmann, M. B. Arniella, M. Holton, S. S. Freeman, V. Naranbhai, C. Stewart, I. Leshchiner, J. Kim, Y. Akiyama, A. T. Griffin, N. I. Vokes, M. Sakhi, V. Kamesan, H. Rizvi, B. Ricciuti, P. M. Forde, V. Anagnostou, J. W. Riess, D. L. Gibbons, N. A. Pennell, V. Velcheti, S. R. Digumarthy, M. Mino-Kenudson, A. Califano, J. V. Heymach, R. S. Herbst, J. R. Brahmer, K. A. Schalper, V. E. Velculescu, B. S. Henick, N. Rizvi, P. A. Jänne, M. M. Awad, A. Chow, B. D. Greenbaum, M. Luksza, A. T. Shaw, J. Wolchok, N. Hacohen, G. Getz, J. F. Gainor, Genomic and transcriptomic analysis of checkpoint blockade response in advanced non-small cell lung cancer. Nat. Genet. 55, 807–819 (2023). 37024582 10.1038/s41588-023-01355-5 PMC10181943

[37] F. Skoulidis, J. V. Heymach, Co-occurring genomic alterations in non-small-cell lung cancer biology and therapy. Nat. Rev. Cancer 19, 495–509 (2019). 31406302 10.1038/s41568-019-0179-8 PMC7043073

[38] S. Spranger, T. F. Gajewski, Impact of oncogenic pathways on evasion of antitumour immune responses. Nat. Rev. Cancer 18, 139–147 (2018). 29326431 10.1038/nrc.2017.117 PMC6685071

[39] N. Ghazali, M. C. Garassino, N. B. Leighl, C. M. Bestvina, Immunotherapy in advanced, *KRAS* G12C-mutant non-small-cell lung cancer: Current strategies and future directions. Ther. Adv. Med. Oncol. 17, 17588359251323985 (2025). 40093982 10.1177/17588359251323985 PMC11907553

[40] F. Skoulidis, L. A. Byers, L. Diao, V. A. Papadimitrakopoulou, P. Tong, J. Izzo, C. Behrens, H. Kadara, E. R. Parra, J. R. Canales, J. Zhang, U. Giri, J. Gudikote, M. A. Cortez, C. Yang, Y. Fan, M. Peyton, L. Girard, K. R. Coombes, C. Toniatti, T. P. Heffernan, M. Choi, G. M. Frampton, V. Miller, J. N. Weinstein, R. S. Herbst, K. K. Wong, J. Zhang, P. Sharma, G. B. Mills, W. K. Hong, J. D. Minna, J. P. Allison, A. Futreal, J. Wang, I. I. Wistuba, J. V. Heymach, Co-occurring genomic alterations define major subsets of KRAS-mutant lung adenocarcinoma with distinct biology, immune profiles, and therapeutic vulnerabilities. Cancer Discov. 5, 860–877 (2015). 26069186 10.1158/2159-8290.CD-14-1236 PMC4527963

[41] F. Skoulidis, M. E. Goldberg, D. M. Greenawalt, M. D. Hellmann, M. M. Awad, J. F. Gainor, A. B. Schrock, R. J. Hartmaier, S. E. Trabucco, L. Gay, S. M. Ali, J. A. Elvin, G. Singal, J. S. Ross, D. Fabrizio, P. M. Szabo, H. Chang, A. Sasson, S. Srinivasan, S. Kirov, J. Szustakowski, P. Vitazka, R. Edwards, J. A. Bufill, N. Sharma, S.-H. I. Ou, N. Peled, D. R. Spigel, H. Rizvi, E. J. Aguilar, B. W. Carter, J. Erasmus, D. F. Halpenny, A. J. Plodkowski, N. M. Long, M. Nishino, W. L. Denning, A. Galan-Cobo, H. Hamdi, T. Hirz, P. Tong, J. Wang, J. Rodriguez-Canales, P. A. Villalobos, E. R. Parra, N. Kalhor, L. M. Sholl, J. L. Sauter, A. A. Jungbluth, M. Mino-Kenudson, R. Azimi, Y. Y. Elamin, J. Zhang, G. C. Leonardi, F. Jiang, K. K. Wong, J. J. Lee, V. A. Papadimitrakopoulou, I. I. Wistuba, V. A. Miller, G. M. Frampton, J. D. Wolchok, A. T. Shaw, P. A. Jänne, P. J. Stephens, C. M. Rudin, W. J. Geese, L. A. Albacker, J. V. Heymach, *STK11*/*LKB1* mutations and PD-1 inhibitor resistance in *KRAS*-mutant lung adenocarcinoma. Cancer Discov. 8, 822–835 (2018). 29773717 10.1158/2159-8290.CD-18-0099 PMC6030433

[42] A. Kolb, J. Kleeff, A. Guweidhi, I. Esposito, N. A. Giese, H. Adwan, T. Giese, M. W. Büchler, M. R. Berger, H. Friess, Osteopontin influences the invasiveness of pancreatic cancer cells and is increased in neoplastic and inflammatory conditions. Cancer Biol. Ther. 4, 740–746 (2005). 15970685 10.4161/cbt.4.7.1821

[43] K. Xu, X. Tian, S. Y. Oh, M. Movassaghi, S. P. Naber, C. Kuperwasser, R. J. Buchsbaum, The fibroblast Tiam1-osteopontin pathway modulates breast cancer invasion and metastasis. Breast Cancer Res. 18, 14 (2016). 26821678 10.1186/s13058-016-0674-8 PMC4730665

[44] J. Qi, H. Sun, Y. Zhang, Z. Wang, Z. Xun, Z. Li, X. Ding, R. Bao, L. Hong, W. Jia, F. Fang, H. Liu, L. Chen, J. Zhong, D. Zou, L. Liu, L. Han, F. Ginhoux, Y. Liu, Y. Ye, B. Su, Single-cell and spatial analysis reveal interaction of *FAP*^+^ fibroblasts and *SPP1*^+^ macrophages in colorectal cancer. Nat. Commun. 13, 1742 (2022). 35365629 10.1038/s41467-022-29366-6 PMC8976074

[45] C. Liu, K. Wu, C. Li, Z. Zhang, P. Zhai, H. Guo, J. Zhang, *SPP1*+ macrophages promote head and neck squamous cell carcinoma progression by secreting TNF-α and IL-1β. J. Exp. Clin. Cancer Res. 43, 332 (2024). 39726047 10.1186/s13046-024-03255-w PMC11670405

[46] C. Lu, Z. Liu, J. D. Klement, D. Yang, A. D. Merting, D. Poschel, T. Albers, J. L. Waller, H. Shi, K. Liu, WDR5-H3K4me3 epigenetic axis regulates OPN expression to compensate PD-L1 function to promote pancreatic cancer immune escape. J. Immunother. Cancer 9, e002624 (2021). 34326167 10.1136/jitc-2021-002624 PMC8323468

[47] X. Su, C. Liang, R. Chen, S. Duan, Deciphering tumor microenvironment: CXCL9 and SPP1 as crucial determinants of tumor-associated macrophage polarity and prognostic indicators. Mol. Cancer 23, 13 (2024). 38217023 10.1186/s12943-023-01931-7 PMC10790255

[48] Y. Chai, H. Xiang, Y. Ma, W. Feng, Z. Jiang, Q. Zhu, Y. Chen, Q. Liu, J. Zhang, J. Ouyang, P. Gao, X. Zhang, S. Chen, L. Jin, H. Lu, S1PR1 suppresses lung adenocarcinoma progression through p-STAT1/miR-30c-5 p/FOXA1 pathway. J. Exp. Clin. Cancer Res. 43, 304 (2024). 39551792 10.1186/s13046-024-03230-5 PMC11571582

[49] G. Orstad, G. Fort, T. J. Parnell, A. Jones, C. Stubben, B. Lohman, K. L. Gillis, W. Orellana, R. Tariq, O. Klingbeil, K. Kaestner, C. R. Vakoc, B. T. Spike, E. L. Snyder, FoxA1 and FoxA2 control growth and cellular identity in NKX2-1-positive lung adenocarcinoma. Dev. Cell 57, 1866–1882.e10 (2022). 35835117 10.1016/j.devcel.2022.06.017 PMC9378547

[50] H. Wang, S. Tang, Q. Wu, Y. He, W. Zhu, X. Xie, Z. Qin, X. Wang, S. Zhou, S. Yao, X. Xu, C. Guo, X. Tong, S. Han, Y. H. Chou, Y. Wang, K. K. Wong, C. G. Yang, L. Chen, L. Hu, H. Ji, Integrative study of lung cancer adeno-to-squamous transition in EGFR TKI resistance identifies RAPGEF3 as a therapeutic target. Natl. Sci. Rev. 11, nwae392 (2024). 39687207 10.1093/nsr/nwae392 PMC11647589

[51] M. B. Amin, F. L. Greene, S. B. Edge, C. C. Compton, J. E. Gershenwald, R. K. Brookland, L. Meyer, D. M. Gress, D. R. Byrd, D. P. Winchester, The Eighth Edition AJCC Cancer Staging Manual: Continuing to build a bridge from a population-based to a more “personalized” approach to cancer staging. CA Cancer J. Clin. 67, 93–99 (2017). 28094848 10.3322/caac.21388

[52] N. A. Rizvi, M. D. Hellmann, A. Snyder, P. Kvistborg, V. Makarov, J. J. Havel, W. Lee, J. Yuan, P. Wong, T. S. Ho, M. L. Miller, N. Rekhtman, A. L. Moreira, F. Ibrahim, C. Bruggeman, B. Gasmi, R. Zappasodi, Y. Maeda, C. Sander, E. B. Garon, T. Merghoub, J. D. Wolchok, T. N. Schumacher, T. A. Chan, Cancer immunology. Mutational landscape determines sensitivity to PD-1 blockade in non-small cell lung cancer. Science 348, 124–128 (2015). 25765070 10.1126/science.aaa1348 PMC4993154

[53] M. D. Hellmann, T. Nathanson, H. Rizvi, B. C. Creelan, F. Sanchez-Vega, A. Ahuja, A. Ni, J. B. Novik, L. M. B. Mangarin, M. Abu-Akeel, C. Liu, J. L. Sauter, N. Rekhtman, E. Chang, M. K. Callahan, J. E. Chaft, M. H. Voss, M. Tenet, X. M. Li, K. Covello, A. Renninger, P. Vitazka, W. J. Geese, H. Borghaei, C. M. Rudin, S. J. Antonia, C. Swanton, J. Hammerbacher, T. Merghoub, N. McGranahan, A. Snyder, J. D. Wolchok, Genomic features of response to combination immunotherapy in patients with advanced non-small-cell lung cancer. Cancer Cell 33, 843–852.e4 (2018). 29657128 10.1016/j.ccell.2018.03.018 PMC5953836

[54] H. Rizvi, F. Sanchez-Vega, K. La, W. Chatila, P. Jonsson, D. Halpenny, A. Plodkowski, N. Long, J. L. Sauter, N. Rekhtman, T. Hollmann, K. A. Schalper, J. F. Gainor, R. Shen, A. Ni, K. C. Arbour, T. Merghoub, J. Wolchok, A. Snyder, J. E. Chaft, M. G. Kris, C. M. Rudin, N. D. Socci, M. F. Berger, B. S. Taylor, A. Zehir, D. B. Solit, M. E. Arcila, M. Ladanyi, G. J. Riely, N. Schultz, M. D. Hellmann, Molecular determinants of response to anti-programmed cell death (PD)-1 and anti-programmed death-ligand 1 (PD-L1) blockade in patients with non-small-cell lung cancer profiled with targeted next-generation sequencing. J. Clin. Oncol. 36, 633–641 (2018). 29337640 10.1200/JCO.2017.75.3384 PMC6075848

[55] R. S. Vanguri, J. Luo, A. T. Aukerman, J. V. Egger, C. J. Fong, N. Horvat, A. Pagano, J. A. B. Araujo-Filho, L. Geneslaw, H. Rizvi, R. Sosa, K. M. Boehm, S. R. Yang, F. M. Bodd, K. Ventura, T. J. Hollmann, M. S. Ginsberg, J. Gao, M. D. Hellmann, J. L. Sauter, S. P. Shah, Multimodal integration of radiology, pathology and genomics for prediction of response to PD-(L)1 blockade in patients with non-small cell lung cancer. Nat. Cancer 3, 1151–1164 (2022). 36038778 10.1038/s43018-022-00416-8 PMC9586871

[56] M. E. Ritchie, B. Phipson, D. Wu, Y. Hu, C. W. Law, W. Shi, G. K. Smyth, limma powers differential expression analyses for RNA-sequencing and microarray studies. Nucleic Acids Res. 43, e47 (2015). 25605792 10.1093/nar/gkv007 PMC4402510

[57] G. Yu, L. G. Wang, Y. Han, Q. Y. He, clusterProfiler: An R package for comparing biological themes among gene clusters. OMICS 16, 284–287 (2012). 22455463 10.1089/omi.2011.0118 PMC3339379

