# Supplementary material for: Mutant p53 sensitizes KRAS-driven lung adenocarcinoma to immunotherapy by repressing SPP1
Source: Sci Adv. 2026 Sep 11;12(37):eaeg9953. doi: 10.1126/sciadv.aeg9953 (PMC13564959; doi:10.1126/sciadv.aeg9953)
Supplement: Supplementary file 1 [file sciadv.aeg9953_sm.pdf]

Science Advances
AAAS

Supplementary Materials for

Mutant p53 sensitizes KRAS-driven lung adenocarcinoma to immunotherapy by repressing SPP1

Xin Zhang *et al.*

Corresponding author: Zhihuang Hu, zhihuanghu@hotmail.com; Xiang Zhou, xiangzhou@fudan.edu.cn; Jialei Wang, wangjialei@shca.org.cn

Sci. Adv. **12**, eaeg9953 (2026)
DOI: 10.1126/sciadv.aeg9953

This PDF file includes:

Figs. S1 to S7
Tables S1 to S4

Figure S1

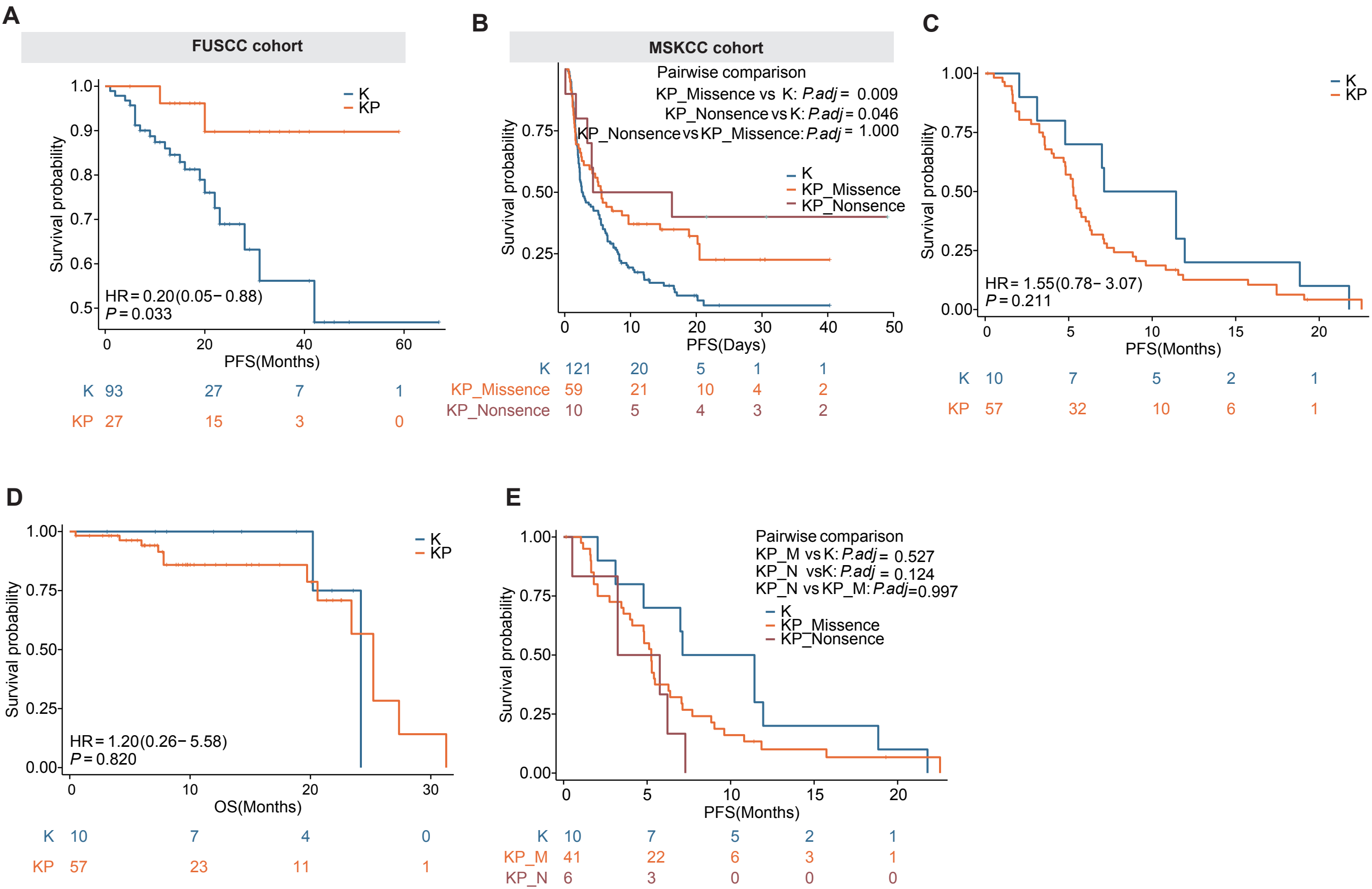

Supplemental Fig1. KRAS/TP53 co-mutation predicts favorable prognosis in immunotherapy-treated LUAD.
(A) Survival analysis of overall survival between KRAS mutation and KRAS/TP53 co-mutation patients in FUSCC cohort (n =120), Statistical significance was determined using two-side Chi-square test. **(B)** Survival analysis of progression-free survival between KRAS mutation, KRAS/TP53 missense mutation and KRAS/TP53 nonsense mutation patients in MSKCC cohort (n =180), Statistical significance was determined using pairwise comparison. **(C-D)** Survival analysis of progression-free survival (C) and overall survival (D) between KRAS mutation and KRAS/TP53 co-mutation patients in a pancreatic cancer immunotherapy cohort (n =67), Statistical significance was determined using two-side Chi-square test. **(E)** Survival analysis of progression-free survival between KRAS mutation, KRAS/TP53 missense mutation and KRAS/TP53 nonsense mutation patients in a pancreatic cancer immunotherapy cohort (n =57), Statistical significance was determined using pairwise comparison.

Figure S2

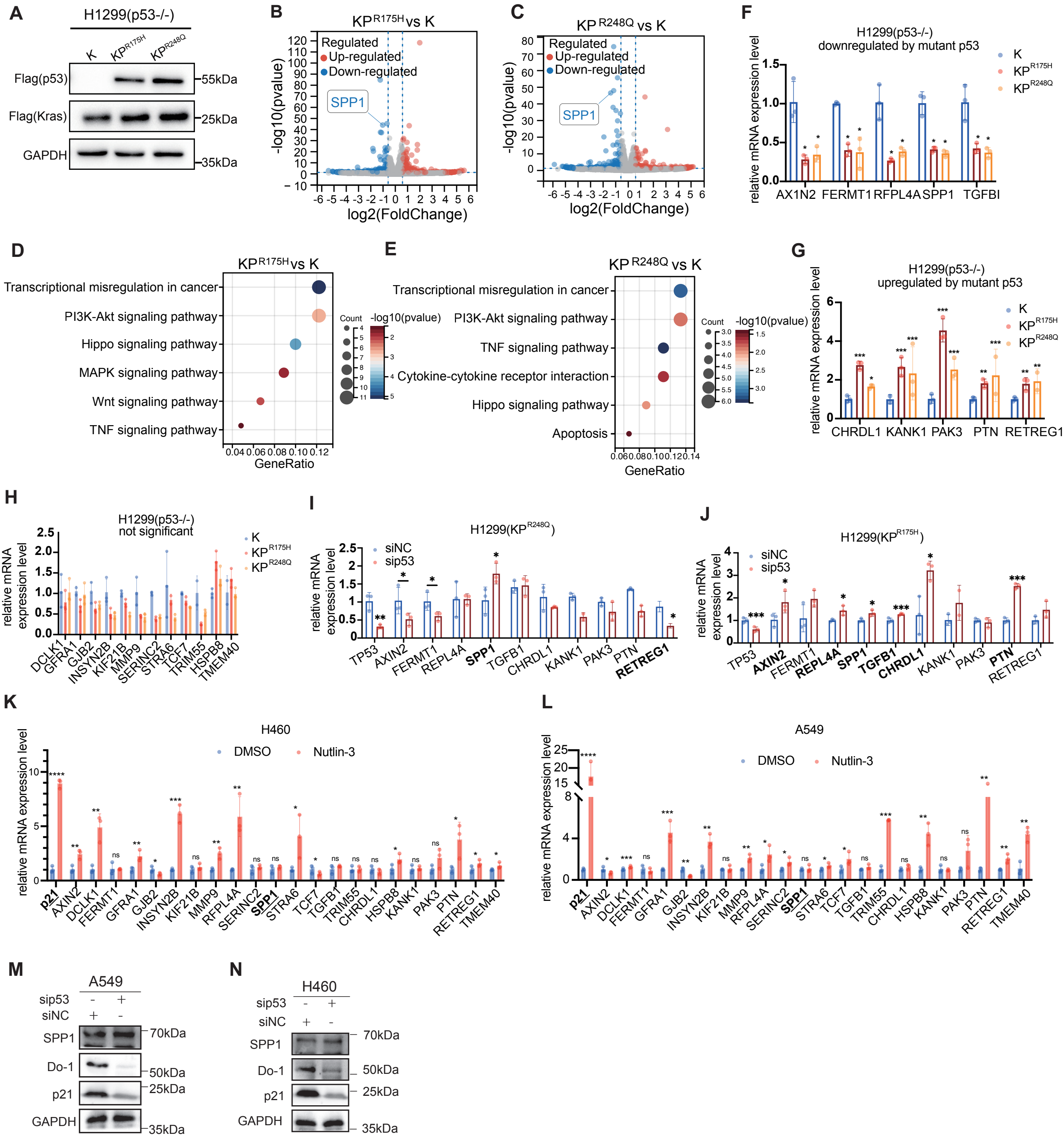

Supplemental Fig2. Identification of SPP1 as a target gene of mutant p53.

(A)WB assay validating the protein expression levels of KRAS^{G12C} mutant, KRAS^{G12C}/TP53^{R175H} and KRAS^{G12C}/TP53^{R248Q} co-mutant isogenic cell lines. **(B-C)** Volcano plot displaying differentially expressed genes (DEGs) between KRAS^{G12C} mutant cells and KRAS^{G12C}/TP53^{R175H} co-mutant cells(B), KRAS^{G12C} mutant cells and KRAS^{G12C}/TP53^{R248Q} co-mutant cells(C). **(D-E)** KEGG pathway enrichment analysis bubble chart of the DEGs identified between KRAS^{G12C} mutant cells and KRAS^{G12C}/TP53^{R175H} co-mutant cells(D), KRAS^{G12C} mutant cells and KRAS^{G12C}/TP53^{R248Q} co-mutant cells(E). **(F-H)** RT-qPCR analysis of SPP1 mRNA expression levels in H1299K cells transfected with Flag-Ctrl, Flag-p53R172H and Flag-p53R270H. **(I-J)** RT-qPCR analyses of SPP1 expression level in KRAS^{G12C}/TP53^{R175H} co-mutant cells (I) and KRAS^{G12C}/TP53^{R175H} co-mutant cells (J) transfected with siNC and sip53. **(K-L)** RT-qPCR analysis of 23 candidate genes mRNA expression levels in H460(K) and A549(L) cells treated with DMSO or Nutlin-3(20uM). **(M-N)** WB assay validating of SPP1 expression level of A549(M) and H460(N) cells transfected with siNC and sip53.

Figure S3

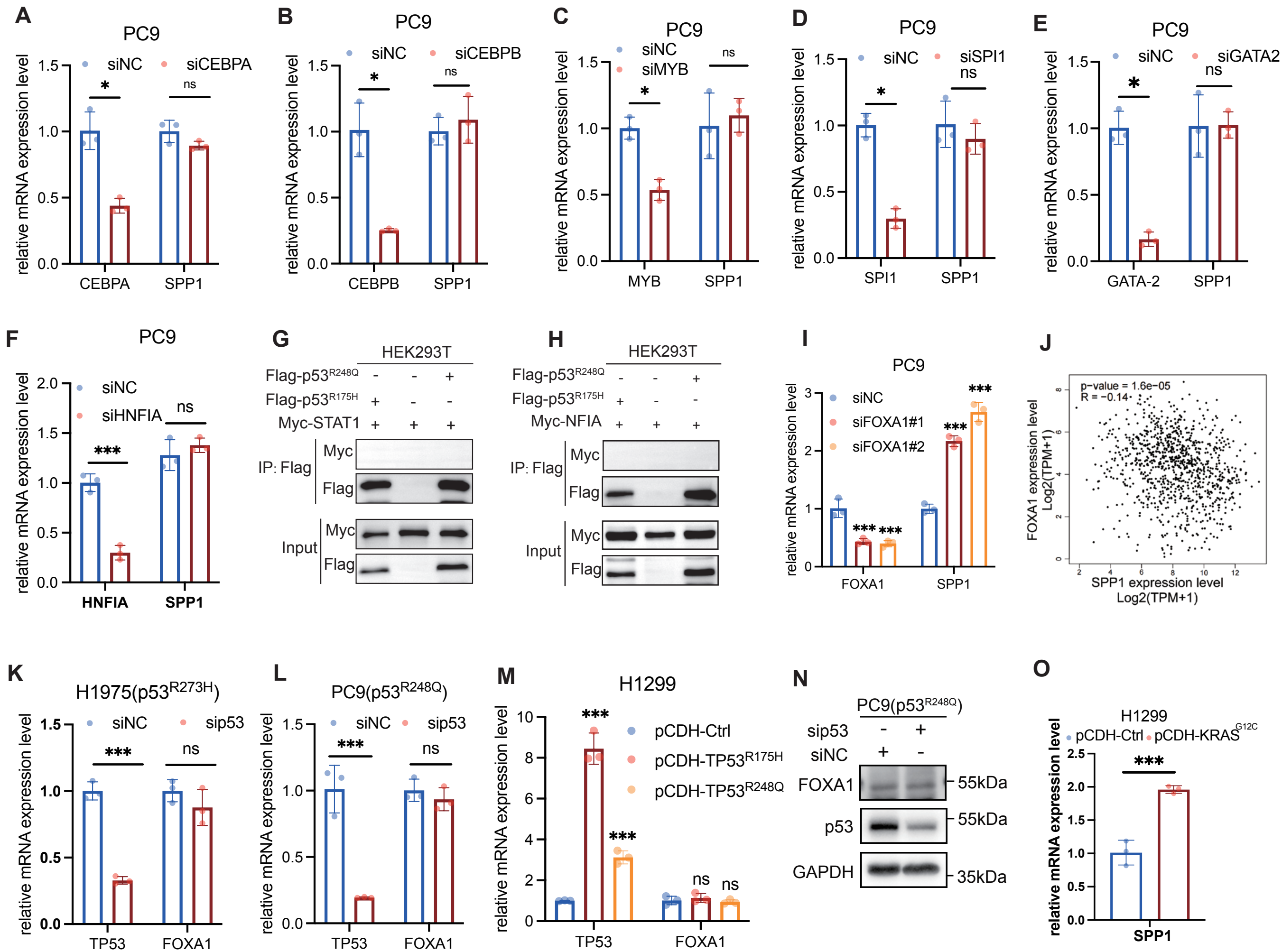

Supplemental Fig3. Mutant p53 cooperates with FOXA1 to repress SPP1 transcription.

(A-F) RT-qPCR analysis of candidate transcription factors (TFs) and SPP1 mRNA levels in PC9 cells transfected with siRNAs targeting CEBPA (A), CEBPB (B), MYB (C), SPI1 (D), GATA2 (E), or HNF1A (F). Data are mean ± SD (n=3), *, P < 0.05, ns, not significant, two-tailed unpaired t-test. **(G-H)** Exogenous co-immunoprecipitation (Co-IP) assay in HEK293T cells co-transfected with Flag-tagged control plasmid or Flag-tagged mutant p53 (p53-R175H or p53-R248Q) and Myc-tagged STAT1 (G) or NFIA (H). Input: total cell lysate; IP: immunoprecipitation with anti-Flag antibody. Western blot (IB) was performed with anti-Myc or anti-Flag antibody. **(I)** RT-qPCR analysis of FOXA1 and SPP1 mRNA levels in PC9 cells transfected with two independent siRNAs targeting FOXA1 (siFOXA1#1, siFOXA1#2). Data are mean ± SD (n=3), ***, P < 0.001, one-way ANOVA. **(J)** Correlation analysis of FOXA1 and SPP1 mRNA expression levels in TCGA-LUAD cohort (n=530). Pearson's correlation coefficient (r) and P-value are indicated. **(K–L)** RT-qPCR analysis of TP53 and FOXA1 mRNA levels in H1975 (K) and PC9 (L) cells transfected with sip53 or siNC. Data are mean ± SD (n=3), ***P < 0.001; ns, not significant, two-tailed unpaired t-test. **(M)** RT-qPCR analysis of TP53 and FOXA1 mRNA levels in H1299 cells (p53-null) transfected with pcDNA3.1-TP53-R175H, pcDNA3.1-TP53-R248Q, or empty vector (pcDNA3.1-Ctrl). Data are mean ± SD (n=3), ***, P < 0.001, ns, not significant, one-way ANOVA. **(N)** Western blot analysis of FOXA1 and p53 protein levels in PC9 cells transfected with sip53 or siNC. **(O)** RT-qPCR analysis of SPP1 mRNA levels in H1299 cells transfected with pcDNA3.1-KRAS-G12C or pcDNA3.1-Ctrl. Data are mean ± SD (n=3), ***P < 0.001, two-tailed unpaired t-test.

Figure S4

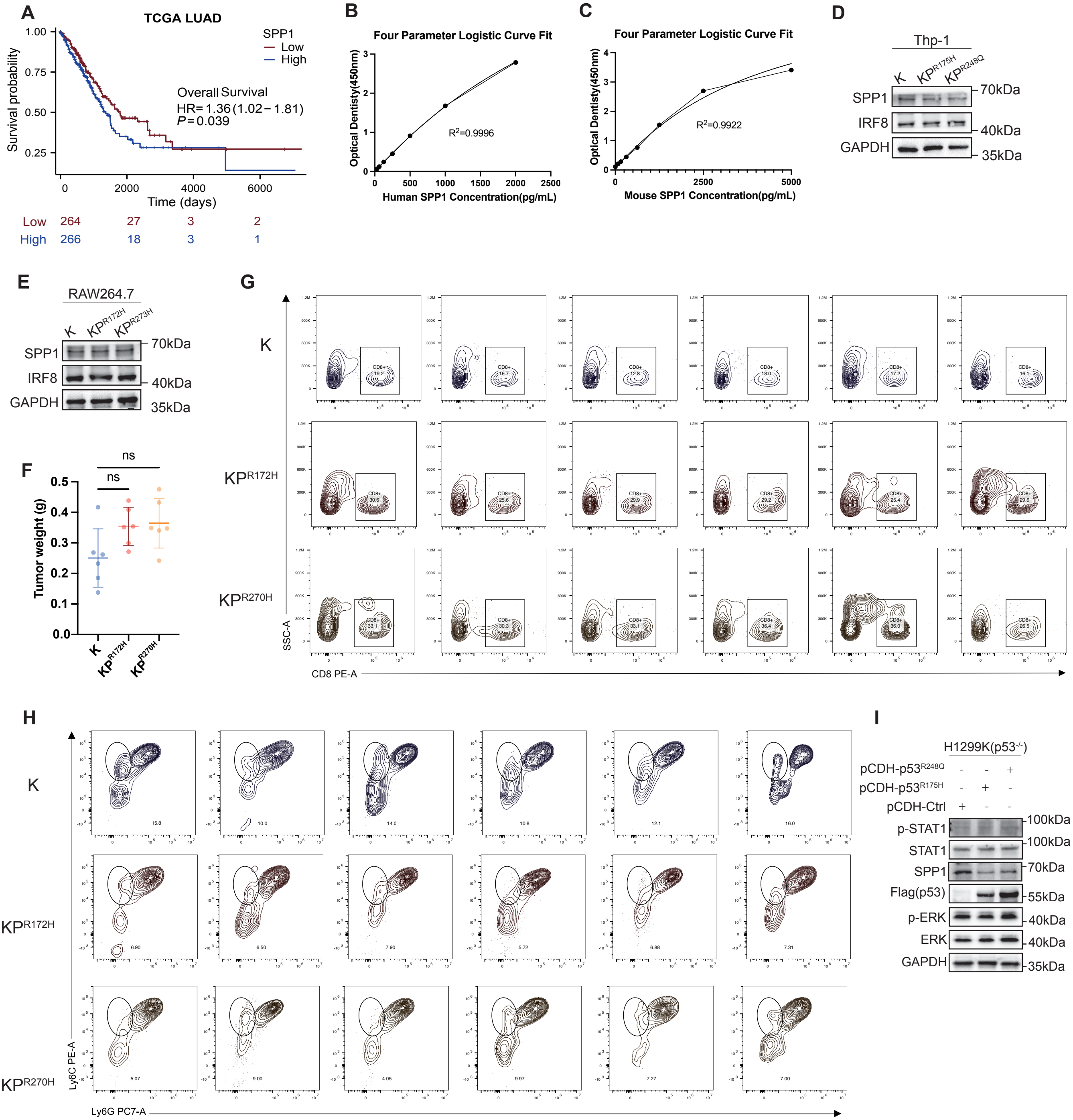

Supplemental Fig4. Mutant p53 impairs monocyte migration by reducing SPP1 secretion.

(A) Survival analysis of overall survival between SPP1 high and low group in TCGA-LUAD cohort (n = 530), Statistical significance was determined using two-side Chi-square test. **(B)** Four parameters logistic (4PL) standard curve for human SPP1 quantification. **(C)** Four parameters logistic (4PL) standard curve of mouse SPP1 quantification. **(D)** Western blot analysis of SPP1 and IRF8 protein expression level in Thp-1 induced by co-culture with H1299K, H1299KP^{R175H} and H1299KP^{R248Q} cells. **(E)** Western blot analysis of SPP1 and IRF8 protein expression level in RAW264.7 induced by co-culture with KP, KP^{R172H} and KP^{R270H} cells. **(F)** Weight quantification of xenograft tumors from KP, KP^{R172H} and KP^{R270H} cells. Data are mean ± SEM (n=6), ns, nonsignificant, one-way ANOVA. **(G-H)** All flow cytometry plots of tumor-infiltrating CD8+ T cells (G) and M-MDSC cells(H) (representative images and quantification corresponding to those in SF4G and H (pannel G: rows 1,col 1; row 2, col 3, and row 3, col 4; pannel H: row 1, col 1; row 2, col 3, and row 3, col 6) are also provided in Figure 4K and L). **(I)** Western blot analysis of p-STAT1, STAT1, SPP1, p-ERK, ERK protein levels in H1299K cells transfected with pCDH-Ctrl, pCDH-p53^{R175H} or pCDH-p53^{R248Q}.

Figure S5

A

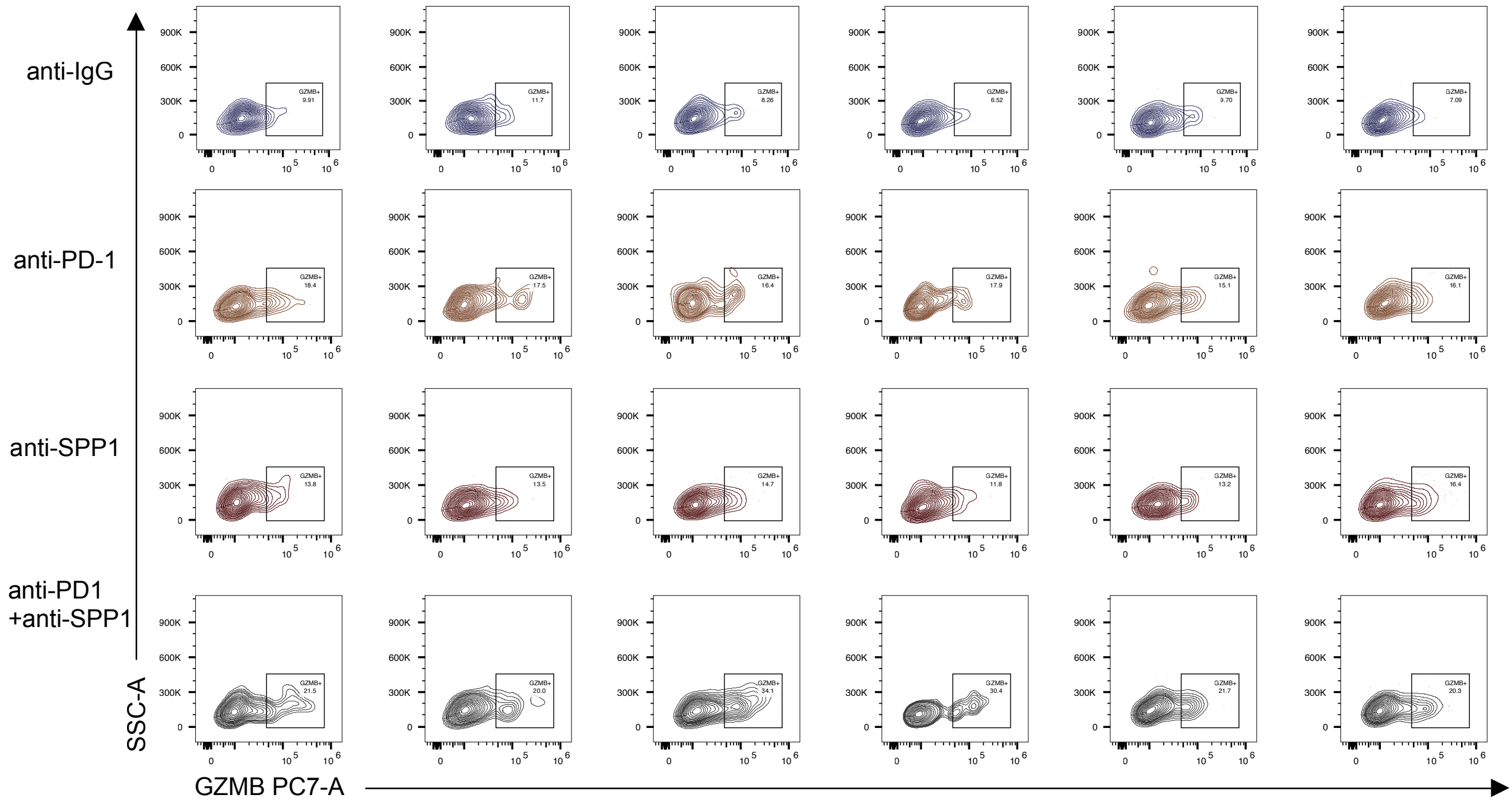

B

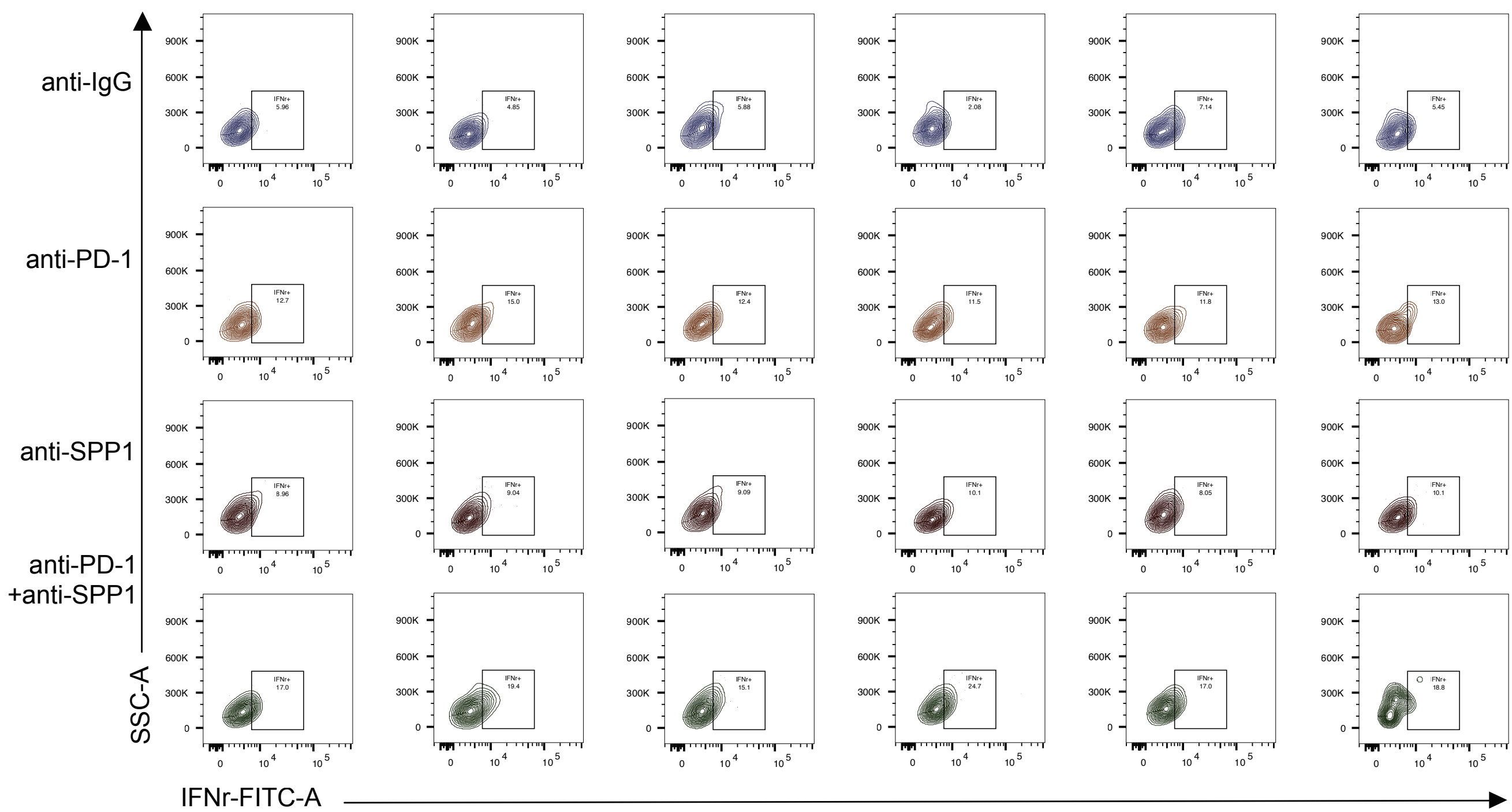

Supplemental Fig5. SPP1 blockade improves anti-PD-1 efficacy in KRAS-driven LUAD.

(A-B) All flow cytometry plots of tumor infiltrating GZMB+CD8+ T cells (A), IFNγ+CD8+ T cells(B) (representative images and quantification corresponding to those in SF5A and B (pannel A: rows 1,col 3; row 2, col 2; row 3, col 1 and row 4, col 5; pannel B: row 1, col 3; row 2, col 3; row 3, col 3 and row 4, col 6) are also provided in Figure 6F and G).

Figure S6

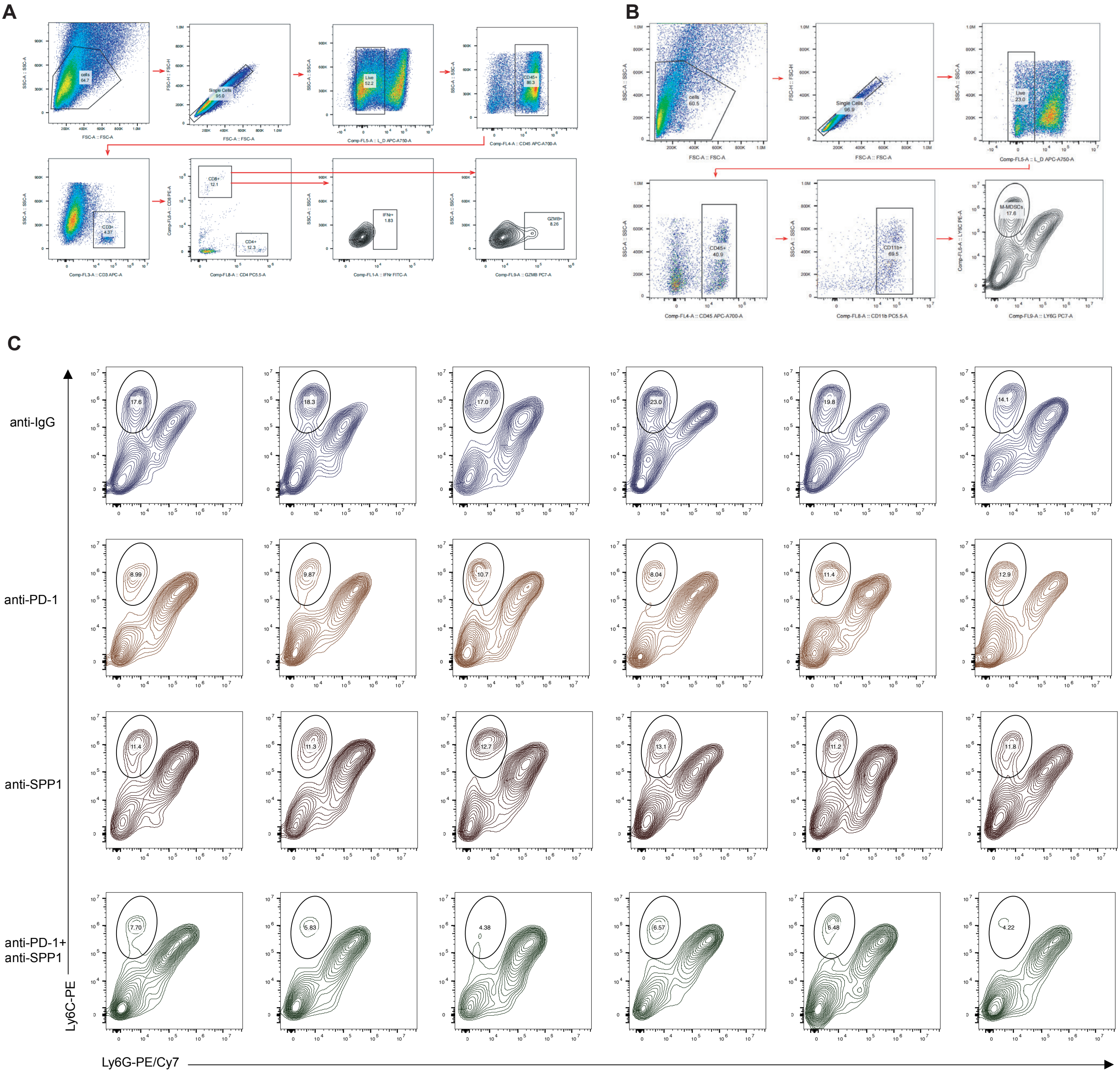

Supplemental Fig6. SPP1 blockade improves anti-PD-1 efficacy in KRAS-driven LUAD.

(A) Represent gating of GZMB+CD8+ T cells and IFNγ+CD8+ T cells. (B) Represent gating of M-MDSCs (Ly6C^{high}Ly6G^{low}). (C) All flow cytometry plots of tumor infiltrating M-MDSCs (Ly6C^{high-} Ly6G^{low}) (representative images and quantification corresponding to those in SF6C (rows 1,col 3; row 2, col 3; row 3, col 2 and row 4, col 2) are also provided in Figure 6H) .

Figure S7

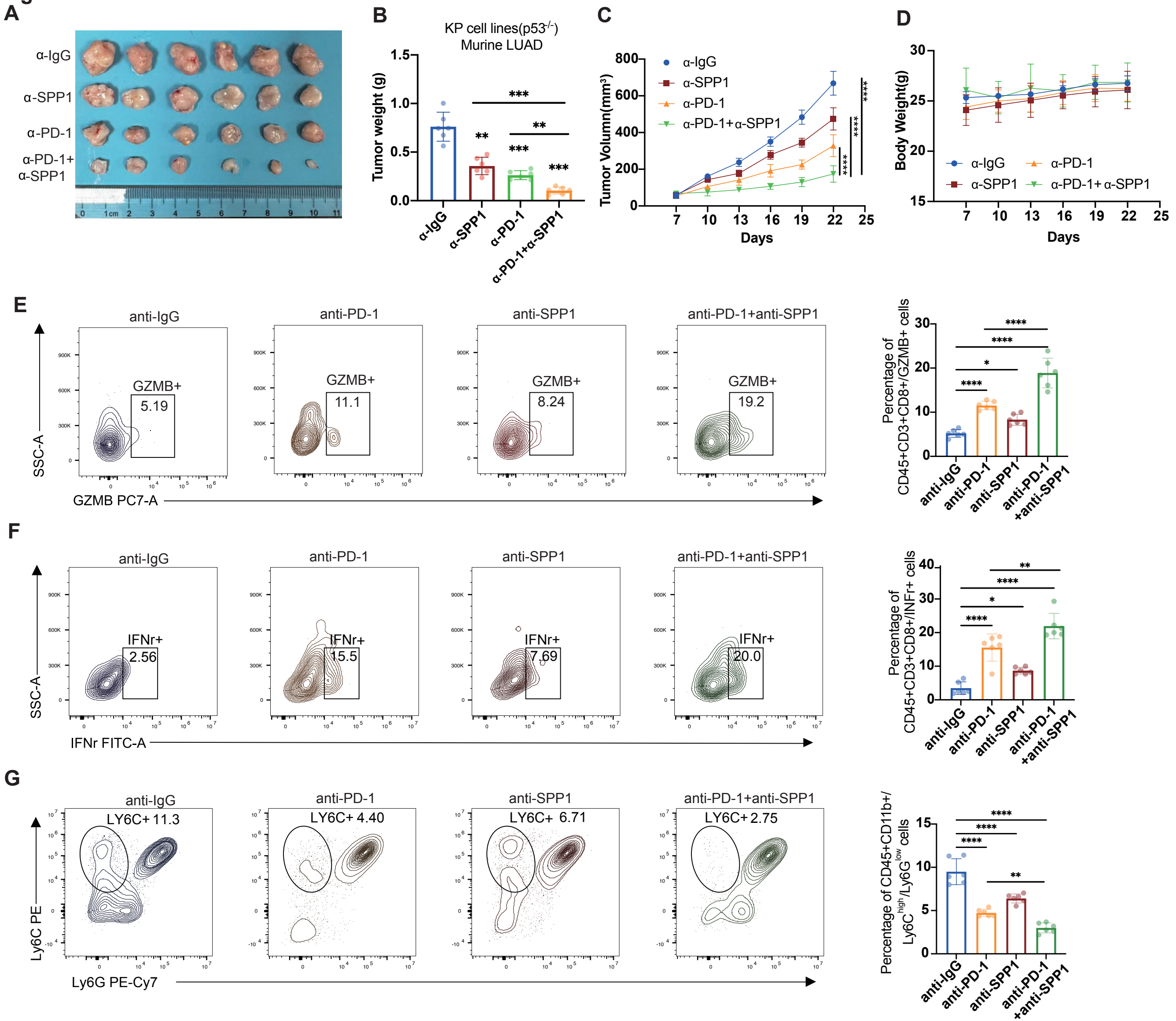

Supplemental Fig7. SPP1 blockade improves anti-PD-1 efficacy in male KRAS-driven LUAD.

(A-C) The size (A), weight (B) and volume (C) of xenograft tumors from each treatment group. **(D)** Mouse body weight kinetics during the treatment period. Data are mean ± SEM (n=6); ns, not significant. **(E-F)** Representative flow cytometry plots (left) and quantification (right) of tumor-infiltrating GZMB+CD8+ T cells (E), IFNγ+CD8+ T cells (F) and M-MDSC cells **(G)** from each treatment group. Data are mean ± SEM (n=6), *, P <0.05, **, P <0.01, ***, P <0.001, ****, P <0.0001, one-way ANOVA.

Supplemental table1. Univariat and multivariat Cox analysis for progression-free survival in LUAD patients.

Characteristics	Total(N)	Univariate analysis		Multivariate analysis	
		Hazard ratio (95% CI)	P value	Hazard ratio (95% CI)	P value
group	120				
K	93	Reference		Reference	
KP	27	0.411 (0.199 - 0.848)	**0.016**	0.411 (0.199 - 0.848)	**0.016**
Gender	120				
male	104	Reference			
female	16	1.256 (0.615 - 2.567)	0.532		
Age	120	1.002 (0.969 - 1.037)	0.890		
Smoking history	120				
never	25	Reference			
previous or current	95	0.641 (0.355 - 1.159)	0.141		
Stage	120				
III	17	Reference			
IV	103	1.107 (0.474 - 2.586)	0.815		
PD-1 inhibitor	120				
Pembrolizumab	60	Reference			
Camrelizumab	8	2.276 (1.025 - 5.055)	**0.043**		
Tislelizumab	43	1.339 (0.719 - 2.493)	0.357		
Sintilimab	6	2.300 (0.683 - 7.741)	0.179		
atezolizumab	3	3.984 (0.925 - 17.155)	0.063		

Characteristics	Total(N)	Univariate analysis		Multivariate analysis	
		Hazard ratio (95% CI)	P value	Hazard ratio (95% CI)	P value
Bone	120				
0	77	Reference			
1	43	1.325 (0.772 - 2.275)	0.307		
Liver	118				
0	110	Reference			
1	8	0.398 (0.097 - 1.636)	0.201		
Brain	117				
0	95	Reference			
1	22	0.555 (0.260 - 1.184)	0.128		
Adrenal gland	119				
0	103	Reference			
1	16	1.381 (0.693 - 2.750)	0.359		
Chest	120				
0	92	Reference			
1	28	1.533 (0.858 - 2.739)	0.149		

Suplemental table 2. The primers used for construction plasmids.

Primer	Sequence（5’-3’）
pcDNA3.1-p53-F	CGCGGATCCATGGAGGAGCCGCAGTCAGA
pcDNA3.1-p53-R	CCGCTCGAGTCAGTCTGAGTCAGGCCCTT
pCDH-KRAS-F	CGGGATCCGATGACTGAATATAAACTTGTGGTAGTTGG
pCDH-KRAS-R	CGCTCGAGTTACATTATAATGCATTTTTTAATTTTCACACAG
SPP1-Luc1-F	GGGGTACCAAAGGAAAAAAATAGTTTTTAGAAG
SPP1-Luc1-R	CCGCTCGAGGGGCAAATCGCCCAG
SPP1-Luc2-F	GGGGTACCCTCAGAAAAAACAACCAGC
SPP1-Luc2-R	CCGCTCGAGTAGATTAAATAATGTCTTGCAG
FOXA1-RE3-del-F	ACAGTAAATCCTGTGGAAATT
FOXA1-RE3-del-R	TGCCCACGGGAATGATTC
FOXA1-RE4-del-F	AGGAAAAAAATAGTTTTTAGAAGCAG
FOXA1-RE4-del-R	ACCCGCATAAGCGTGTCAT
FOXA1-RE5-del-F	GAAACATCCCAAAACATGACCTCC
FOXA1-RE5-del-R	AAACATGCTGCTGCTGGTTG
FOXA1-RE6-del-F	CGGGTCATTGTTGGGAAAT
FOXA1-RE6-del-R	TTGTCTCCTTCCCGACTTCTACA

Supplemental table 3. siRNA sequences

siRNA	Sequences（5’-3’)
siNC	UUCUCCGAACGUGUCACGUTT
sip53	GUAAUCUACUGGGACGGAATT
siFOXA1#1	CCGUCCUAAACACUUCCUATT
siFOXA1#2	CACUGCAAUACUCGCCUUATT
siSTAT1	CAGGAAGACCCAAUCCAGAUGUCUA
siNFIA	GGAGGUUGGACCUUGUUAUTT
siCEBPA	CAGAGAGCUCCUUGGUCAAGGCCAU
siCEBPB	CCCUGCGGAACUUGUUCAAGCAGCU
siGATA2	ACUAGUGUCCGCAUAAGAAAAAGAA
siSPI1	GAGAGCUUCGCCGAGAACAACUUCA
siMYB	GAGACACUAUAAUGAUGAAGA

Supplemental table4. Primer sequences

Primer	Sequence (5'-3')
GAPDH-F	GGAGCGAGATCCCTCCAAAAT
GAPDH-R	GGCTGTTGTCATACTTCTCATGG
p53-F	CAGCACATGACGGAGGTTGT
p53-R	TCATCCAAATACTCCACACGC
KRAS-F	ACAGAGAGTGGAGGATGCTTT
KRAS-R	TTTCACACAGCCAGGAGTCTT
AXIN2-F	TACACTCCTTATTGGGCGATCA
AXIN2-R	TTGGCTACTCGTAAAGTTTTGGT
DCLK1-F	GCTGATTTGACCCGAACTCTG
DCLK1-R	AGCCACATACATAACTCTCTCCT
FERMT1-F	CTTGTTAAAGCCGTCTGGTGA
FERMT1-R	ACTGAACCAAGTCATGGTGGAT
GFRA1-F	CCAAAGGGAACAACTGCCTG
GFRA1-R	CGGTTGCAGACATCGTTGGA
GJB2-F	ACATCGAGGAGATCAAAACCCA
GJB2-R	GAAGCCGTCGTACATGACATAG
INSYN2B-F	AGATGGGACCACTAAGAATCCA
INSYN2B-R	CTGGCTGTGAGTCTCTTCCTT
KIF21B-F	ACCTATGACTTTGTCTTCGACCT
KIF21B-R	CAGCACCGTGGCATTATAGC
MMP9-F	TGTACCGCTATGGTTACACTCG
MMP9-R	GGCAGGGACAGTTGCTTCT
RFPL4A-F	CAGTGCCTCAATTCACTCCAG
RFPL4A-R	TCCACATCGAACGTCATATCCA
SERINC2-F	GCCTTTGACAACGAGCAGGA
SERINC2-R	CCAGTTGGTGAGCGTCATCA
SPP1-F	CTCCATTGACTCGAACGACTC
SPP1-R	CAGGTCTGCGAAACTTCTTAGAT
STRA6-F	CCACAGAGGACTACTCCTATGG
STRA6-R	CAGCACAAGGATTGACAGCG
TCF7-F	TGCACATGCAGCTATACCCAG
TCF7-R	TGGTGGATTCTTGGTGCTTTTC
TGFBI-F	CTTCGCCCCTAGCAACGAG

TGFBI-R	TGAGGGTCATGCCGTGTTTC
TRIM55-F	TTGTCAGCACAACCTGTGTAG
TRIM55-R	CCCATGTCTATCCAAAACCACTT
CHRDL1-F	CCTGGAACCTTATGGGTTGGT
CHRDL1-R	AACATTTGGACATCTGACTCGG
HSPB8-F	CTCCTGCCACTACCCAAGC
HSPB8-R	GGCCAAGAGGCTGTCAAGT
KANK1-F	GTGCCGAGGAGAACATGAAC
KANK1-R	CTCTAAGCTGTACTTCTAGGCGA
PAK3-F	CCAGGCTTCGCTCTATCTTCC
PAK3-R	TCAAACCCCACATGAATCGTATG
PTN-F	GGAGCTGAGTGCAAGCAAAC
PTN-R	CTCGCTTCAGACTTCCAGTTC
RETREG1-F	CTCAGCCACTGTATTGCAGAA
RETREG1-R	AACAATGGACACAAAAATGCACA
TMEM40-F	CAGAGCAACCGGAAAACATCG
TMEM40-R	TCATCCTTCAAAACGTCAGGC
CD70-F	GCTTTGGTCCCATTGGTCG
CD70-R	CGTCCCACCCAAGTGACTC
HBE1-F	ATGGTGCATTTTACTGCTGAGG
HBE1-R	GGGAGACGACAGGTTTCCAAA
TUSC1-F	GCCTCTTCCGTCAGGCTTT
TUSC1-R	CGGAGTCGGGTTCCTGTAGA
ZCCHC24-F	CGAGGGCCTGACTCCATAC
ZCCHC24-R	ACACGTTGATGTGGCACTTGA
